# Immersive metaverse art as a psychological intervention for depression and anxiety: a narrative review and multilevel model integrating engagement, cultural identity, and neurocognitive mechanisms

**DOI:** 10.3389/fpsyg.2026.1829947

**Published:** 2026-06-03

**Authors:** Si Chen, Verly Veto Vermol, Jiarui Yu, Huizhi Jiang

**Affiliations:** 1 College of Creative Art, Universiti Teknologi MARA, Shah Alam, Selangor, Malaysia; 2 School of Art and Design Jewelry, Baoshan University, Baoshan, Yunnan, China; 3 School of Fashion Art and Design, Wuxi Vocational Institute of Arts & Technology, Yixing, Jiangsu, China

**Keywords:** anxiety, avatar, cultural identity, depression, digital intervention, flow, immersive art therapy, metaverse

## Abstract

Depression and anxiety rank among the leading causes of global disability, yet traditional treatments reach only a minority of affected individuals. The COVID-19 pandemic further exacerbated this crisis, triggering a ~ 25% worldwide surge in anxiety and depression prevalence. In parallel, immersive digital environments (the “metaverse”) are maturing as platforms for creative expression and social connection. This review proposes that immersive metaverse art – interactive art experiences in VR/AR – can act as a multilevel psychobehavioral modulator. We integrate recent evidence showing that such experiences enhance engagement (flow, presence), enable identity exploration (customizable avatars, cultural narratives), and engage neurocognitive systems (reward, attention, regulation). Empirical studies of VR art interventions report acute mood improvements, stress reduction, and greater social connectedness. We synthesize these findings into a conceptual model linking core components (immersion, creative agency, social avatar, cultural symbolism) to mediating processes (flow, meaning-making, belonging) and outcomes (symptom relief, emotional regulation, behavioral activation). Rather than examining these domains as separate interdisciplinary themes, the review integrates them within a unified clinical framework focused on transdiagnostic mechanisms relevant to depression and anxiety. We compare immersive art therapy with traditional art therapy, noting unique advantages (scalability, personalization) and novel risks (overdependence, identity diffusion). Finally, we outline translational pathways – e.g. integrating VR art with cognitive therapies and highlight the need for rigorous trials and cross-cultural validation. Overall, immersive metaverse art emerges as a promising, if nascent, approach to mental health intervention, warranting further empirical and ethical scrutiny.

## 1 Introduction: immersive metaverse art as a psychobehavioral modulator

### 1.1 The global burden of depression and anxiety in the digital era

Depressive and anxiety disorders are major contributors to global disability, with post-pandemic estimates showing sustained elevations compared to pre-2020 baselines (Liu and Kuai, 2025; Liu Y. et al., 2025). Global burden analyses indicate ongoing increases in prevalence and YLDs, especially among adolescents and young adults immersed in digital ecosystems (Liu Y. et al., 2025; Fan et al., 2025). Heavy digital engagement—prolonged screen time, algorithmic content, and social comparison—is linked to heightened depressive and anxiety symptoms (Godard and Holtzman, 2023; Marciano et al., 2024). Meta-analyses show small-to-moderate associations between passive social media use and affective symptoms (Marciano et al., 2024).

Mechanistic studies suggest that digital overstimulation and algorithm-driven exposure contribute to emotional dysregulation and cognitive reactivity (Yue et al., 2022). Experimental paradigms indicate that emotionally valenced digital feeds acutely alter mood and physiological arousal, moderated by baseline psychopathology, usage, and context (Yue et al., 2022). Digital environments thus actively modulate attentional and affective processes.

The global mental health treatment gap remains large; most individuals with depression or anxiety lack adequate care, exacerbated by socioeconomic and geographic disparities (Acuña-Rodríguez et al., 2025). Even in high-income countries, workforce shortages, cost, and waitlists constrain therapy (Barbui et al., 2025; Chisholm et al., 2016). Dropout rates in CBT range 20–35%, influenced by symptom severity and logistical barriers (Fernandez et al., 2015). Digital interventions, including internet-delivered CBT, improve scalability and cost-effectiveness (Zainal et al., 2024), but engagement and adherence, especially in unguided formats, remain challenges (Kambeitz-Ilankovic et al., 2022). Consequently, process-based models targeting shared mechanisms such as rumination and motivational deficits are increasingly emphasized (Moskow et al., 2023). Immersive technologies are now being explored as direct modulators of psychological processes.

### 1.2 From digital consumption to digital intervention

Originally designed for entertainment, VR has expanded into clinical domains including exposure therapy, pain modulation, and affective skills training (Li Pira et al., 2023; Ding et al., 2025). Passive digital consumption typically involves observation with limited interactivity, whereas immersive therapeutic environments feature presence, agency, and structured tasks. Presence—the sense of “being there”—correlates with emotional engagement and therapeutic responsiveness (Augustin et al., 2024; Moon et al., 2025). Interactive immersion enables feedback loops enhancing attention and perceived control.

RCTs show short-term symptom reduction for depression and anxiety when guidance is included (Zeng et al., 2025); While structured immersive interventions show therapeutic promise, immersion driven by escapism motives has been associated with increased psychological distress and maladaptive coping patterns in vulnerable individuals (Jouhki et al., 2022; Gursesli et al., 2025). Immersive artistic participation emphasizes creative agency, symbolic expression, and narrative engagement. Therapeutic design principles include autonomy-supportive tasks, culturally resonant symbolism, adaptive challenges for flow, and safety protocols (Lu and Li, 2026). User agency correlates with improved self-efficacy and affective outcomes (Paul et al., 2024). However, high-intensity multisensory stimulation may increase cognitive load when interactive and perceptual demands exceed regulatory capacity (Dai et al., 2025), and immersion motivated by avoidance tendencies may reinforce maladaptive coping trajectories (Jouhki et al., 2022; Gursesli et al., 2025). The field must shift VR from hedonic platforms to evidence-based psychobehavioral modulators.

### 1.3 Conceptual positioning of the review

This review integrates the broad interdisciplinary scope of immersive metaverse art practices, engagement principles from engagement psychology, cultural identity frameworks, neurocognitive mechanisms, and clinical translational research into a unified multilevel model. Immersive metaverse art functions as a unifying platform in which technological affordances simultaneously drive high engagement (flow and presence), facilitate culturally resonant identity exploration through customizable avatars and symbolic narratives, modulate key neurocognitive processes of reward processing, attentional reorientation, prefrontal-limbic regulation, and social cognition, and thereby enable scalable clinical interventions for depression and anxiety. The integrative logic across these domains is that psychological mediators (engagement and identity) are instantiated in and reinforced by neurocognitive substrates, which in turn underpin transdiagnostic mechanisms and support concrete clinical translation; this interdependent architecture offers a more comprehensive explanation than singular mechanisms and clarifies how the domains converge to produce therapeutic effects.

#### 1.3.1 Engagement pathway

Immersive absorption reduces rumination, especially when inducing flow, which enhances mood and diminishes cognitive intrusions (Ma et al., 2025; Ma et al., 2025). Attentional reallocation aligns with process-based psychopathology models (Moskow et al., 2023). Self-determination theory suggests that autonomy, competence, and relatedness foster intrinsic motivation and resilience (Chakrabarti, 2025).

#### 1.3.2 Identity pathway

Avatar-mediated identity exploration (“Proteus effect”) influences self-perception, self-efficacy, and narrative reframing (Martin Coesel et al., 2024; Liu, 2025). Culturally embedded symbolism enhances belonging, while narrative identity frameworks allow rehearsal of alternative self-stories to strengthen coherence and regulation (Ellis et al., 2022; McAdams and McLean, 2013).

#### 1.3.3 Neurocognitive pathway

Neuroimaging shows VR engages striatal reward circuits and prefrontal cognitive control regions (Lu et al., 2025a), modulating prefrontal–limbic connectivity and attenuating default mode network activity, consistent with reduced rumination (Chen et al., 2025; Lee et al., 2021). Social avatar interaction recruits mentalizing networks (Li Pira et al., 2023; Xue et al., 2024). Evidence mainly reflects acute activation; durable neuroplastic changes are still limited (Li Pira et al., 2023). A multilevel framework integrating engagement, identity, and neurocognition offers a more comprehensive explanation than singular mechanisms (Moskow et al., 2023).

### 1.4 Methodological approach

This manuscript constitutes a narrative review that synthesizes existing literature and proposes a novel multilevel conceptual model of immersive metaverse art as a psychobehavioral intervention for depression and anxiety. Narrative synthesis was selected given the emerging, interdisciplinary, and heterogeneous nature of research on immersive technologies and mental health, allowing integration of empirical, mechanistic, and theoretical insights to advance conceptual understanding rather than exhaustive quantification of effects.

In constructing this review, we conducted a narrative synthesis of literature from 2020 and 2026, focusing on original studies, meta-analyses, and theoretical papers relevant to VR/AR art and mental health. Databases (e.g., PubMed, PsycINFO, IEEE Xplore) were searched for terms such as “virtual reality art therapy”, “immersive experience depression”, “avatar self-experimentation”, and “metaverse mental health”. Inclusion criteria included peer-reviewed English-language publications (empirical studies, meta-analyses, and theoretical/conceptual papers) examining immersive (VR/AR/metaverse) artistic or creative experiences in relation to psychological processes (e.g., presence, flow, avatar embodiment, cultural symbolism) or clinical applications for depression and anxiety. Exclusion criteria encompassed non-peer-reviewed sources, studies not involving immersive art or creative components, publications prior to 2020 (except for foundational theoretical references), and those lacking relevance to the specified psychological or clinical outcomes.

We prioritized empirical findings (e.g., RCTs, experimental trials, physiological studies) and mechanistic insights (e.g., neuroimaging, computational models) in accordance with evidence hierarchies favoring higher-quality designs such as randomized controlled trials and meta-analyses over observational or conceptual studies, while incorporating the latter to capture emerging interdisciplinary perspectives and to support the development of the proposed conceptual model (Hadjipanayi et al., 2023). Emerging technologies (AI-driven VR, biosensors) and sociocultural analyses (digital art and identity) were also surveyed to inform future directions. Because the field is evolving rapidly, we emphasized the most recent and high-quality sources. We did not perform a systematic meta-analysis due to the heterogeneity of methods, but aimed for a comprehensive narrative that aligns with the outlined conceptual framework. Throughout, we have highlighted key references to support major assertions.

## 2 Conceptual framework: a multilevel model of immersive art intervention

**Figure 1:**
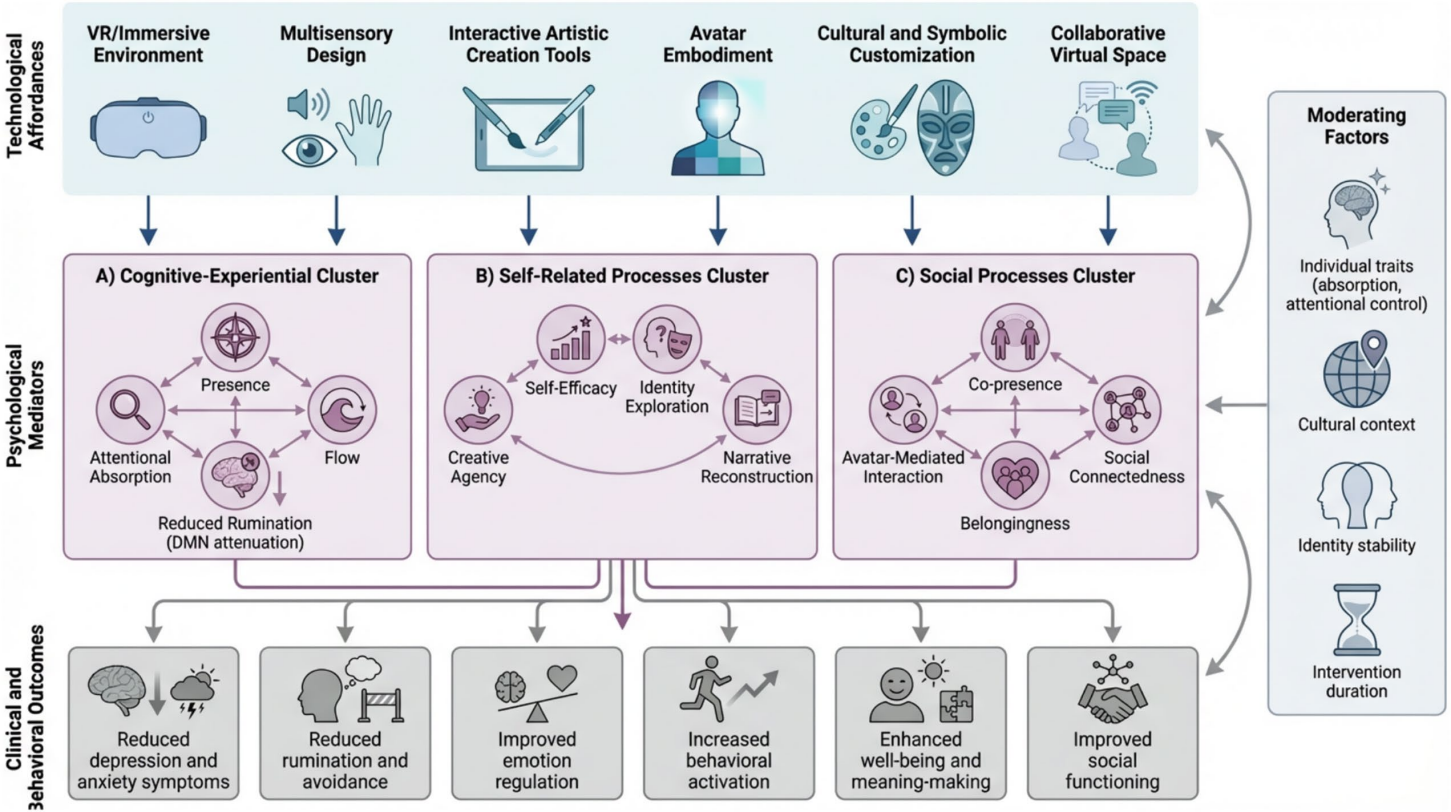
Multilevel conceptual framework linking immersive metaverse art to psychological and clinical outcomes. Immersive artistic environments provide a set of technological affordances—including virtual reality environments, multisensory design, interactive creation tools, avatar embodiment, cultural customization, and collaborative virtual spaces—that engage multiple clusters of psychological mediators. These mediators operate across three interrelated domains: cognitive-experiential processes (e.g., attentional absorption, presence, and flow), self-related processes (e.g., creative agency, self-efficacy, identity exploration, and narrative reconstruction), and social processes (e.g., co-presence, avatar-mediated interaction, and social connectedness). Through these pathways, immersive engagement may attenuate maladaptive rumination, enhance emotion regulation, promote behavioral activation, and strengthen belongingness and meaning-making. The model further highlights moderating influences—including individual traits, cultural context, identity stability, and intervention duration—that may shape responsiveness to immersive interventions and their downstream effects on depression, anxiety, and social functioning.

Immersive metaverse art interventions can be conceptualized as multilevel systems in which technological affordances, psychological mediators, and clinical outcomes interact dynamically rather than linearly. This framework integrates environmental design features, intrapsychic and interpersonal mediators, and downstream transdiagnostic effects, offering a structured lens to evaluate both experiential and clinical dimensions of digital artistic engagement. Figure 1 illustrates the proposed multilevel framework linking technological affordances of immersive artistic environments with psychological mediators and downstream clinical outcomes.

Although this multilevel framework draws upon and integrates established theoretical models—including Self-Determination Theory (autonomy, competence, relatedness) (Chakrabarti, 2025), process-based psychotherapy approaches (Moskow et al., 2023), transdiagnostic models of rumination and avoidance (Jingili et al., 2023; Lee et al., 2024), and the Proteus effect in avatar research (Martin Coesel et al., 2024; Liu, 2025)—it provides a structured synthesis tailored to immersive metaverse art interventions. Unlike prior VR therapeutic models, which have predominantly focused on exposure-based mechanisms, relaxation, or general mindfulness applications in virtual environments (Li Pira et al., 2023; Zeng et al., 2025; Lee et al., 2024), the present model emphasizes the interplay of creative agency, cultural symbolism, and avatar-mediated identity exploration within artistic creation contexts. By positioning technological affordances of metaverse art (multisensory immersion, interactive tools, customizable cultural narratives) as drivers engaging multiple mediator clusters simultaneously, this framework extends existing single-mechanism accounts toward a more integrated account of immersive artistic engagement and its potential therapeutic effects on depression and anxiety symptoms. Thus, its novelty lies not in proposing entirely new psychological constructs, but in synthesizing and extending existing theories into a domain-specific architecture tailored to immersive metaverse art interventions.

### 2.1 Core components

Preliminary evidence from experimental studies suggests that head-mounted immersive artistic environments can elicit higher subjective presence than non-immersive 2D digital media, with medium-to-large effect sizes reported in several laboratory paradigms (Souza et al., 2022; Barranco Merino et al., 2023). Presence is commonly measured using validated instruments such as the Igroup Presence Questionnaire (IPQ), Slater–Usoh–Steed (SUS) scale, and Presence Questionnaire (PQ), all of which exhibit adequate internal consistency and construct validity within mental health samples (Barranco Merino et al., 2023; Grassini and Laumann, 2020). Neurocognitive correlates of heightened presence include increased activation in sensorimotor integration networks and modulation of medial prefrontal regions associated with self-referential processing (Vessel et al., 2013). Preliminary fMRI findings suggest that immersive artistic engagement attenuates default mode network (DMN) activity relative to non-immersive viewing, potentially reflecting reductions in maladaptive rumination (Vessel et al., 2013). Sensory congruence—the alignment of visual, auditory, and embodied interaction cues— has been shown to predicts depth of immersion, with multisensory synchrony enhancing both presence and emotional salience (Lyu et al., 2023). Individual differences moderate immersion intensity; trait absorption and attentional control predict stronger presence experiences, whereas dissociation proneness may intensify immersion while increasing susceptibility to maladaptive detachment (McDonald et al., 2025). Socially shared immersive experiences generally produce higher co-presence but slightly reduced self-focused absorption compared to solitary immersion, indicating differential cognitive allocation between interpersonal attunement and introspective engagement (Moreira Kares et al., 2025).

Active artistic creation within immersive environments has been associated with greater psychological benefits than passive exposure in controlled comparisons. Controlled comparisons of interactive digital painting or sculpting in VR versus passive viewing have reported improvements in positive affect and perceived competence (Guerra-Tamez, 2023; Yang X. et al., 2024). Perceived creative control enhances intrinsic motivation, consistent with self-determination theory’s emphasis on autonomy and competence satisfaction (Grasse et al., 2022). Mechanistically, creative agency supports self-efficacy by providing immediate feedback loops linking action and outcome, with neurobehavioral evidence indicating that goal-directed creative interaction engages reward circuitry more strongly than passive observation (Lehrman, 2025). Small-scale follow-up studies suggest that repeated immersive creative engagement over several weeks may yield sustained improvements in mood and self-efficacy, though durability beyond 3 months remains underexplored (Ding et al., 2025). Customization of digital artistic elements, including color palettes, symbolic forms, and avatar embodiment, predicts higher autonomy satisfaction and engagement persistence (Yang X. et al., 2024); however, excessive complexity or unstructured creative freedom can induce cognitive overload. Optimal design requires adaptive scaffolding that balances user agency with structured guidance (Guerra-Tamez, 2023).

Avatar-mediated interaction is a defining feature of immersive metaverse art platforms. Evidence indicates that embodied avatars can enhance perceived social connectedness compared to text-based or non-embodied interaction (Moreira Kares et al., 2025). The “Proteus effect” literature demonstrates that avatar embodiment can influence behavior, perspective-taking, and empathy, particularly in collaborative artistic tasks (Fusaro et al., 2025). Customization features enhance psychological safety and self-disclosure, particularly when anonymity reduces fear of negative evaluation (Van Brakel et al., 2023). For socially anxious individuals, anonymous avatars often facilitate initial engagement, whereas identity-linked avatars may strengthen accountability and relational continuity (Van Brakel et al., 2023). Perceived co-presence, measured via social presence scales, predicts emotional engagement and collaborative satisfaction during shared immersive artistic creation (Son et al., 2025). Nevertheless, high-frequency avatar use carries potential risks, including identity fragmentation and self-concept instability, especially among adolescents with pre-existing identity diffusion (Aardema et al., 2010).

Immersive art is not psychologically neutral; symbolic content shapes engagement and meaning-making. Culturally familiar artistic motifs enhance emotional resonance and perceived relevance compared to culturally neutral environments (Gong et al., 2025). Narrative coherence—defined as the integration of personal and cultural themes within immersive storytelling—predicts existential meaning and identity affirmation (Lin et al., 2025). Cross-cultural studies reveal substantial variation in the interpretation of immersive symbolism, indicating that culturally embedded design must be context-sensitive (Lin et al., 2025). Comparative evidence suggests that culturally resonant immersive art experiences strengthen belongingness and self-esteem among minority participants more effectively than generic environments, while representation of marginalized identities may enhance psychological safety and social inclusion (Gong et al., 2025).

### 2.2 Mediating processes

Flow states, defined by an optimal balance between challenge and skill, can arise during immersive artistic engagement (Guerra-Tamez, 2023). Experimental work has shown that VR-based creative tasks calibrated for adaptive difficulty may increase flow and reduce self-reported rumination relative to non-interactive controls (Yang X. et al., 2024). Neurophysiological studies associate immersive absorption with modulation of attentional networks and decreased DMN activation, consistent with reduced self-referential processing (Vessel et al., 2013). Design variables, including task complexity, interactivity, and real-time feedback, predict flow likelihood (Yang X. et al., 2024). Evidence for sustained benefits beyond individual sessions is emerging but remains limited, with repeated engagement appearing necessary for cumulative effects (Buragohain et al., 2025).

Immersive artistic environments also facilitate narrative reconstruction by enabling symbolic self-expression and role experimentation (Lin et al., 2025). Avatar-based identity exploration has been associated with increased self-concept clarity when guided by reflective prompts, although unguided experimentation may yield ambiguity in some users (Lin et al., 2025). Existential and narrative identity frameworks suggest that symbolic interaction supports meaning-making by externalizing internal conflicts into manipulable artistic forms (Vessel et al., 2013; Lin et al., 2025). Preliminary findings indicate modest increases in identity flexibility following multi-session immersive programs (Wang Y. et al., 2025), moderated by baseline identity stability, cultural congruence of symbolism, and availability of structured reflection.

Collaborative immersive art experiences have been associated with perceived social inclusion, measured via belongingness and social connectedness scales (Van Brakel et al., 2023; Son et al., 2025). Belongingness mediates reductions in depressive and anxiety symptoms in several digital intervention studies (Van Brakel et al., 2023). Synchronous artistic activities, such as real-time co-creation, generate stronger bonding than asynchronous participation due to temporal alignment and shared attentional focus (Son et al., 2025). Evidence suggests that these effects can modestly generalize to offline social functioning, although longitudinal confirmation remains limited (Baschab et al., 2026). Structured collaborative tasks appear more effective than unstructured interactions in promoting inclusive engagement (Moreira Kares et al., 2025). The core technological and experiential components of immersive metaverse art interventions, together with the primary psychological mediators through which they exert influence, are synthesized in Table 1.

**Table 1:** Core Components and Psychological Mediators in Immersive Metaverse Art Interventions.

Level / Category	Key component / Feature	Description / Primary effects	Moderators / Design influences	Associated mediating processes	Reference(s)
Technological / Experiential	Heightened subjective presence	Medium–large effect vs. 2D media; increased sensorimotor integration, DMN attenuation	Trait absorption, attentional control, dissociation proneness	Reduced rumination, enhanced emotional salience	Souza et al. (2022), Barranco Merino et al. (2023), Grassini and Laumann (2020), Vessel et al. (2013), Lyu et al. (2023), and McDonald et al. (2025)
Technological / Experiential	Multisensory congruence and sensory synchrony	Predicts immersion depth and emotional intensity	Alignment of visual, auditory, embodied cues	Increased presence and emotional engagement	Lyu et al. (2023)
Technological / Experiential	Active artistic creation vs. passive exposure	Greater positive affect, perceived competence, intrinsic motivation	Adaptive scaffolding, task complexity	Creative agency, autonomy and competence satisfaction	Guerra-Tamez (2023), Yang X. et al. (2024), Grasse et al. (2022), and Lehrman (2025)
Technological / Experiential	Customization and creative control	Enhances autonomy, engagement persistence; risk of overload if unstructured	Color/symbol choice, avatar embodiment	Self-efficacy, intrinsic motivation	Yang X. et al. (2024) and Grasse et al. (2022)
Social / Interpersonal	Embodied avatar-mediated interaction	Enhanced social connectedness, co-presence; Proteus effect on empathy & perspective-taking	Anonymity vs. identity-linked avatars	Perceived social support, belongingness	Moreira Kares et al. (2025), Fusaro et al. (2025), Van Brakel et al. (2023), and Son et al. (2025)
Social / Interpersonal	Collaborative / shared immersive experiences	Higher co-presence; stronger bonding in synchronous co-creation	Temporal alignment, structured vs. unstructured tasks	Social inclusion, emotional engagement	Moreira Kares et al. (2025), Van Brakel et al. (2023), and Son et al. (2025)
Symbolic / Cultural	Culturally resonant motifs and narrative coherence	Increased emotional resonance, meaning-making, belongingness	Cultural familiarity, representation of identities	Existential meaning, identity affirmation	Gong et al. (2025) and Lin et al. (2025)
Psychological State	Flow state	Optimal challenge–skill balance; reduced rumination	Adaptive difficulty, real-time feedback, interactivity	Attentional absorption, decreased self-referential processing	Guerra-Tamez (2023), Yang X. et al. (2024), and Buragohain et al. (2025)
Psychological state	Narrative reconstruction and identity exploration	Symbolic self-expression, role experimentation; modest increases in identity flexibility	Guided reflection, baseline identity stability	Self-concept clarity, meaning-making	Lin et al. (2025) and Wang Y. et al. (2025)
Psychological state	Belongingness and social connectedness	Mediates symptom reduction in collaborative contexts	Synchronous interaction, perceived co-presence	Reduced depressive/anxiety symptoms	Van Brakel et al. (2023), Son et al. (2025), and Baschab et al. (2026)

### 2.3 Downstream outcomes

Controlled trials indicate that immersive artistic interventions reduce transdiagnostic symptom clusters, particularly rumination, avoidance, and anhedonia (Ma et al., 2025). Compared with conventional app-based interventions, immersive art platforms often achieve comparable short-term symptom reduction but higher engagement and experiential intensity (Ma et al., 2025). Mediation analyses implicate flow, agency satisfaction, and belongingness as statistical pathways of symptom improvement (Ma et al., 2025; Van Brakel et al., 2023). Maintenance beyond one month remains inconsistently documented.

Immersive artistic engagement enhances cognitive reappraisal capacity by enabling experiential reframing within symbolic environments (Lin et al., 2025). Physiological markers, including heart rate variability and skin conductance, show modest reductions in autonomic arousal during immersive sessions (Ma et al., 2025). Evidence supports attentional redirection and meaning-making as complementary mechanisms for reducing negative affect, with neuroimaging suggesting potential top-down modulation of limbic reactivity via prefrontal engagement (Vessel et al., 2013).

Creative accomplishment within immersive environments reinforces goal-directed behavior through reward-based feedback loops (Lehrman, 2025). Pilot studies report increased real-world activity following immersive art participation, consistent with behavioral activation principles (Ma et al., 2025). Social or system-generated feedback enhances persistence and engagement (Yang X. et al., 2024). While evidence for sustained behavioral activation beyond novelty effects is limited, repeated structured engagement appears promising for mitigating withdrawal in depressed populations (Paul et al., 2022).

## 3 Engagement and identity as psychological mediators

Immersive metaverse art exerts psychological effects through interrelated pathways involving attentional engagement, identity exploration, and culturally mediated meaning-making. By integrating multisensory immersion, interactive creativity, and symbolic narrative structures, these environments can interrupt maladaptive cognitive cycles, enhance emotion regulation, and promote adaptive behavioral activation. This section synthesizes empirical findings concerning attentional, hedonic, eudaimonic, avatar-mediated, and cultural mechanisms, while delineating moderating variables and boundary conditions that shape therapeutic impact.

### 3.1 Presence, flow, and attentional reorientation

Psychological presence—the subjective sense of “being there” within a mediated environment—constitutes a foundational mechanism of immersive interventions. Contemporary research operationalizes presence using validated instruments such as the Igroup Presence Questionnaire (IPQ) and the Presence Questionnaire (PQ), which assess spatial immersion, involvement, and ecological validity (Mostajeran et al., 2023; Schwind et al., 2019). Empirical studies indicate that elevated perceived presence in immersive artistic contexts correlates with reductions in depressive rumination, suggesting that attentional redirection mediates affective improvement (Ma et al., 2025; Lem et al., 2024). Multisensory congruence—including synchronized visual, auditory, and haptic inputs—enhances perceptual coherence and deepens absorption, thereby strengthening emotional regulation outcomes. Importantly, trait-level moderators—including absorption proneness, baseline attentional control, and dissociative sensitivity—significantly influence immersion intensity and downstream psychological effects (Simonian et al., 2025; Hayes and Campagnoli, 2026).

Sustained engagement depends on a calibrated balance between environmental realism and user agency. Relative to non-immersive digital media, immersive systems reallocate attentional resources from default-mode self-referential processing toward externally oriented task engagement, thereby attenuating maladaptive rumination (Wienrich et al., 2021). Experimental paradigms employing eye-tracking, dual-task interference, and attentional network testing confirm measurable shifts in cognitive allocation during immersive participation, with patterns paralleling attentional redistribution observed in mindfulness-based interventions (Chiossi et al., 2025; Mikhailenko et al., 2022). These findings support the proposition that immersive art can function as a structured attentional retraining context rather than merely a distraction mechanism.

Flow represents a related but distinct construct characterized by an optimal challenge–skill balance, deep concentration, and intrinsic reward. Interactive immersive art frequently induces flow states when task complexity is dynamically calibrated to user capability. Empirical studies demonstrate that flow reduces repetitive negative thinking and enhances affective resilience among individuals with depressive and anxiety symptoms (Moore and Butler, 2024; Guerra-Tamez, 2025). Mechanistically, flow mediates outcomes through intensified intrinsic motivation, anticipatory reward signaling, and sustained attentional focus, allowing users to experience competence and mastery within a psychologically safe virtual space (Bieri et al., 2025). Task calibration is critical: insufficient complexity yields boredom and disengagement, whereas excessive cognitive demand generates frustration and attrition (Ma et al., 2025).

Neurocognitive investigations link flow states to coordinated activation within frontoparietal attentional networks and striatal dopaminergic circuitry, reflecting the integration of cognitive control and reward processing (Gold and Ciorciari, 2021; Gold and Ciorciari, 2020). Complementary EEG and fMRI studies demonstrate attenuation of default mode network (DMN) activity during immersive artistic engagement, paralleling neural signatures observed in mindfulness meditation and associated with reduced self-referential rumination (Van Schouwenburg et al., 2012; Klasen et al., 2012). Experimental paradigms confirm that attentional redeployment mediates acute symptom reduction, with dual-task performance and eye-tracking data demonstrating immediate shifts in cognitive focus (Israel et al., 2019). Repeated exposure combined with reflective prompts or scaffolded challenges appears to extend benefits beyond single sessions; however, durability is moderated by baseline rumination severity, immersion intensity, and session duration (Pu and Luo, 2025; Ma et al., 2023).

Collectively, presence and flow operate as attentional regulators that temporarily disrupt maladaptive self-referential loops, potentially creating a window for cognitive restructuring and behavioral activation.

### 3.2 Hedonic versus eudaimonic digital experiences

Immersive metaverse art encompasses both hedonic and eudaimonic experiential dimensions. Hedonic engagement emphasizes sensory pleasure, novelty, and immediate affective gratification, whereas eudaimonic engagement prioritizes purpose, self-development, and meaning-making (Rieger et al., 2014). Meaning-centered interaction is measured using validated instruments such as the Digital Eudaimonia Scale and experience-sampling methodologies capturing reflective, goal-directed participation (Daneels et al., 2021).

Empirical comparisons indicate that hedonic immersion reliably produces short-term mood elevation and stress reduction; however, these effects are typically transient (Mostajeran et al., 2023). In contrast, eudaimonic engagement—characterized by narrative coherence, intentional creativity, and reflective depth—predicts more sustained reductions in depressive symptoms and greater behavioral activation over time (Seaborn et al., 2020; Peiró et al., 2019). The mediating construct of perceived personal growth appears central: immersive experiences that foster competence, agency, and identity integration reinforce self-efficacy and promote durable motivational change (Su et al., 2020). Purely hedonic experiences, although pleasurable, rarely generate enduring cognitive restructuring or sustained goal-directed behavior.

Autonomy-supportive design amplifies eudaimonic outcomes. Users who perceive alignment between their personal values and immersive content exhibit higher intrinsic motivation, greater persistence, and enhanced well-being gains (Huta, 2016; Joshanloo, 2016). Platform scaffolding—through narrative prompts, collaborative creation, and structured reflection—facilitates the transition from passive hedonic consumption to purpose-driven engagement. These findings underscore the importance of intentional design architecture in transforming immersive art from entertainment to psychologically constructive intervention.

### 3.3 Avatar-mediated self-expansion

Avatar embodiment constitutes a distinctive mechanism within immersive metaverse environments. Avatars provide a controlled medium for exploring alternative or aspirational identities, thereby enhancing self-concept clarity and attenuating distress (Yee and Bailenson, 2007). Customization of physical attributes, expressive behaviors, and narrative roles enables experimentation with suppressed or idealized traits. Longitudinal engagement, particularly when paired with structured reflection, promotes identity flexibility and adaptive self-representation (Lin et al., 2021). Controlled studies report reductions in anxiety and depressive symptoms following temporary identity experimentation, suggesting that immersive self-reconstruction may facilitate cognitive reframing and self-efficacy enhancement (Dupraz et al., 2024).

Perceived agency within avatar-mediated environments strengthens mastery beliefs. Skill acquisition, problem-solving, and creative production within virtual contexts generalize to increased offline behavioral confidence, consistent with self-efficacy theory (Aymerich-Franch et al., 2014). Embodied perspective-taking further amplifies empowerment by fostering internalization of positive self-representations and promoting prosocial orientation (Delangle et al., 2026). Individuals with low self-esteem or social anxiety frequently leverage avatar-mediated anonymity to initiate social interaction and creative collaboration, circumventing offline inhibition barriers (Yamashita and Yamamoto, 2024).

However, identity experimentation is not uniformly beneficial. Discrepancies between virtual and offline identities may generate cognitive dissonance or fragmentation, particularly when virtual representations diverge markedly from core self-concepts. Empirical findings suggest that perceived authenticity and continuity moderate outcomes: when avatars are experienced as extensions of the self, engagement supports adaptive self-expansion; when perceived as disconnected personas, risks of identity diffusion increase (Jang and Yang, 2026; Kim et al., 2023). Thus, avatar-mediated mechanisms require careful calibration to balance flexibility with coherence.

### 3.4 Cultural symbolism and narrative coherence

Culturally congruent immersive art enhances psychological safety, engagement depth, and perceived belongingness (Vasudha and Choudhary, 2026). Environments incorporating collective memory, shared narratives, and culturally resonant symbolism elicit stronger emotional responses and identity affirmation compared to culturally neutral or ambiguous settings (Garcia-Gutierrez et al., 2025; Gualano et al., 2024). Perceived representation of one’s cultural identity fosters social connectedness and group cohesion, mechanisms central to buffering depressive and anxiety symptoms.

Digital co-creation of culturally meaningful art further strengthens interpersonal bonds and social integration. Social identity theory provides a theoretical framework: immersive participation within culturally congruent narratives reinforces collective identity, which mediates improvements in both online and offline social functioning (Rieger et al., 2014; Daneels et al., 2021). These effects are particularly salient in diasporic or marginalized populations for whom digital environments may serve as sites of identity preservation and affirmation.

Narrative coherence constitutes an additional therapeutic mechanism. Immersive storytelling combined with symbolic artistic engagement enables users to reinterpret adversity, construct alternative life narratives, and cultivate post-traumatic growth (Georgieva and Georgiev, 2019). Empirical assessments using validated measures of meaning in life and identity integration demonstrate that immersive narrative reconstruction predicts improvements in existential well-being and emotional regulation (Ma et al., 2023; Botella et al., 2017). Through guided symbolic interaction, participants can transform fragmented experiences into coherent self-stories, reinforcing psychological integration.

### 3.5 Boundary conditions

Despite documented benefits, immersive engagement is subject to significant boundary conditions. Prolonged or high-intensity exposure may induce technostress, cognitive fatigue, or emotional overload (Luo et al., 2016). Individual differences—including baseline anxiety, attentional capacity, dissociation vulnerability, and prior digital exposure—modulate susceptibility to adverse effects (Saredakis et al., 2020). High sensory density elevates cognitive load and may disrupt attentional absorption, undermining emotional benefits (Chang et al., 2020).

Excessive immersion can also shift adaptive absorption into maladaptive avoidance or escapism. When immersive engagement replaces rather than complements offline coping strategies, symptom relief may prove short-lived or counterproductive. Design safeguards—such as paced session structuring, adaptive difficulty calibration, reflective debriefing prompts, and real-time monitoring of physiological or behavioral indicators—mitigate these risks (Lundin et al., 2023; Kourtesis et al., 2024).

Cultural expectations and sociocontextual factors further moderate identity experimentation outcomes. Normative beliefs regarding self-expression, collectivism, or digital representation influence user comfort and engagement patterns (Cerri et al., 2024). Consequently, context-sensitive design and culturally responsive frameworks are essential to maximize benefit and minimize unintended consequences.

In summary, immersive metaverse art functions as a multilevel psychological mediator integrating attentional regulation, motivational processes, identity flexibility, and cultural meaning-making. Presence and flow reorient attention away from maladaptive rumination; hedonic and eudaimonic pathways differentially shape short- and long-term outcomes; avatar-mediated exploration promotes self-efficacy and adaptive identity reconstruction; and culturally embedded narratives enhance belonging and existential coherence. These mechanisms operate synergistically yet remain contingent on intensity, individual differences, and design parameters. Careful calibration is therefore essential to optimize therapeutic impact across depressive and anxiety-related symptom domains.

Although the preceding sections examine engagement, identity exploration, and cultural meaning-making as distinct psychological dimensions of immersive metaverse art, these processes are unlikely to operate independently in clinical contexts. Rather, immersive artistic environments appear to engage interconnected attentional, affective, motivational, and social systems that collectively shape emotional regulation and behavioral adaptation. To strengthen conceptual integration across the review, the following section examines the neurocognitive substrates that may underlie and connect these experiential and psychological processes, providing a mechanistic bridge between immersive engagement and downstream clinical outcomes.

## 4 Neurocognitive substrates of immersive artistic engagement

**Figure 2:**
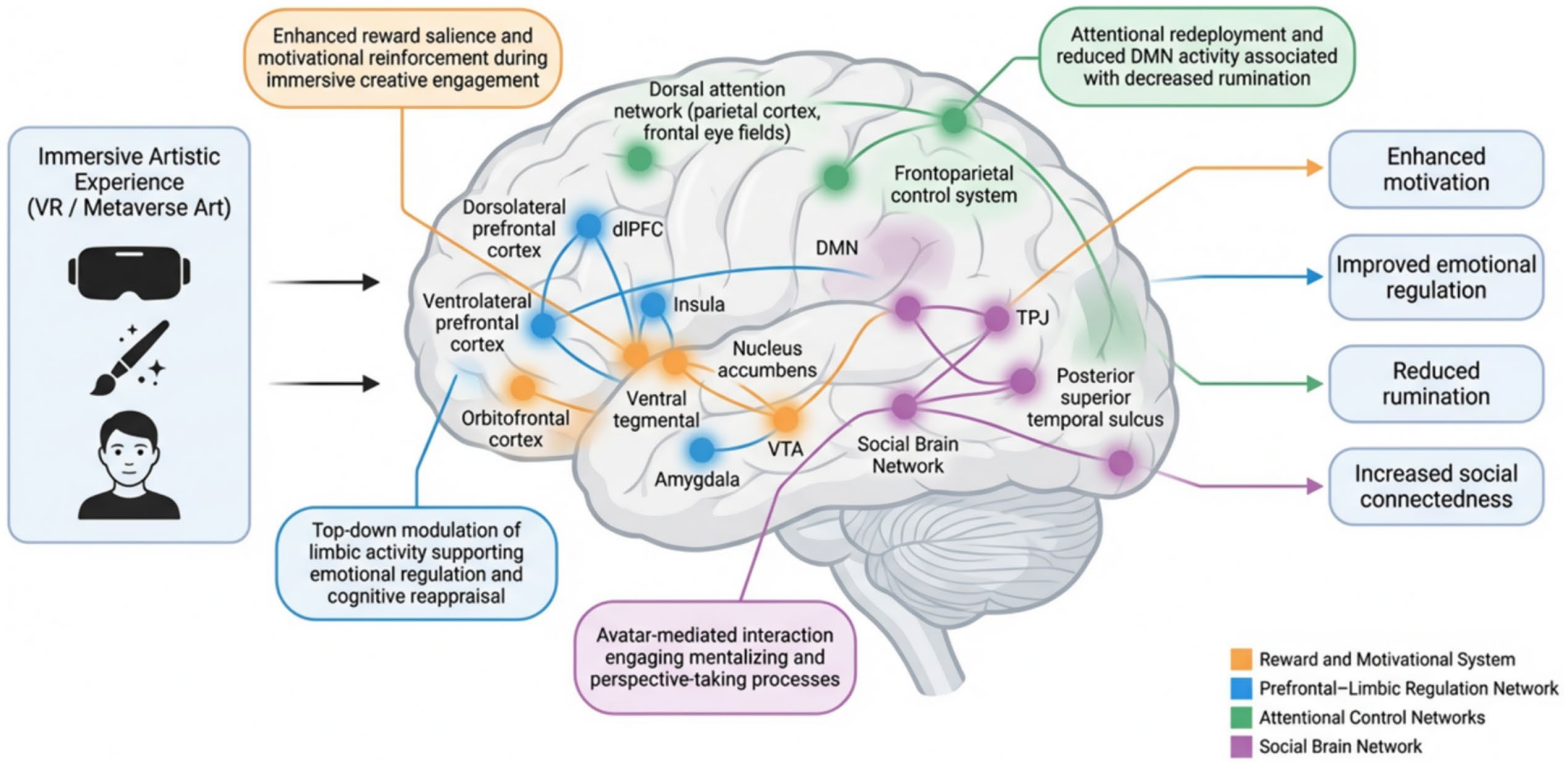
Neurocognitive networks engaged during immersive artistic experience. Immersive artistic engagement in virtual environments recruits multiple neurocognitive systems related to reward processing, emotional regulation, attentional control, and social cognition. Key regions include reward circuitry (VTA and nucleus accumbens), prefrontal–limbic regulatory pathways, attentional control networks associated with reduced default mode activity, and social brain regions involved in perspective taking and connectedness. These interacting systems are proposed to contribute to enhanced motivation, improved emotion regulation, reduced rumination, and increased social connectedness.

Elucidating the neurocognitive mechanisms underlying immersive artistic engagement is essential for determining how such interventions influence reward processing, emotional regulation, attentional control, and social cognition. Recent research increasingly integrates functional neuroimaging, psychophysiological monitoring, and computational modeling to characterize activity within dopaminergic, prefrontal–limbic, attentional, and social brain networks (Figure 2). At present, most findings are based on small-to-moderate sample sizes and short-term designs, requiring cautious interpretation. This section synthesizes empirical evidence, emphasizing mechanistic insights while acknowledging methodological constraints.

### 4.1 Reward and motivational circuitry

Human neuroimaging studies have reported that immersive virtual reality (VR) experiences can recruit reward-related regions, including the ventral striatum and broader mesolimbic pathways (Monaco et al., 2026; Auerbach et al., 2022). Functional magnetic resonance imaging (fMRI) and positron emission tomography (PET) studies—although indirect in their inference of dopaminergic transmission—have shown increased blood-oxygen-level-dependent (BOLD) responses in the nucleus accumbens and ventral tegmental area during active engagement with immersive artistic tasks (Singer et al., 2023; Tang et al., 2022). Experimental designs frequently compare interactive VR conditions with passive media exposure, consistently showing that self-directed exploration and creative agency enhance reward salience, reflected in amplified striatal activation (Lenormand and Piolino, 2022; Van Timmeren et al., 2023).

This pattern is clinically relevant in the context of depression, where blunted reward sensitivity and diminished approach motivation are core features. Individuals with depressive symptoms exhibit attenuated ventral striatal responses to reward cues; repeated immersive exposure has been associated with partial normalization of these responses, suggesting a potential mechanism for ameliorating anhedonia (Ren et al., 2024; Dehghani et al., 2023). Behavioral indices of approach motivation—such as persistence in goal-directed VR tasks and voluntary re-engagement—correlate with neural reward markers, reinforcing the link between immersive artistic participation and motivational reinforcement processes (Cybinski et al., 2024).

However, several methodological limitations constrain interpretation. Most studies employ modest sample sizes (typically *N* = 15–40), limiting statistical power and generalizability. Dopaminergic engagement is inferred from hemodynamic or metabolic proxies rather than direct neurotransmitter measurement. Additionally, novelty effects may transiently amplify reward-related activation, complicating attribution of sustained motivational change to immersive content per se. Controlled comparisons with traditional behavioral activation protocols suggest that immersive artistic engagement elicits comparable striatal recruitment while adding multisensory and agency-mediated components that may enhance experiential salience (Int-Veen et al., 2025; Goodpaster et al., 2026). Nonetheless, disentangling novelty-driven reward from mechanism-specific activation remains a priority for future research.

### 4.2 Prefrontal–limbic modulation and emotional regulation

Immersive environments have been found in some studies to facilitate top-down modulation of limbic structures, particularly the amygdala and insula, through engagement of prefrontal regulatory systems (Mugnol-Ugarte et al., 2024). fMRI investigations report increased activation in the dorsolateral and ventrolateral prefrontal cortex during immersive artistic tasks, paralleling neural signatures associated with cognitive reappraisal and executive control processes targeted in cognitive behavioral therapy (CBT) (Wang L. et al., 2025; Tang et al., 2025). Concurrent attenuation of amygdala reactivity during emotionally evocative VR content has been observed, with effect magnitudes comparable to established emotion regulation paradigms (Park et al., 2022).

Psychophysiological indices provide convergent support. Measures such as heart rate variability (HRV) and skin conductance demonstrate enhanced autonomic flexibility and reduced sympathetic arousal during immersive engagement, particularly among individuals with elevated baseline anxiety (Jeanne et al., 2025; Catalogna et al., 2025a). Increased HRV during emotionally salient VR tasks suggests improved vagal regulation, consistent with enhanced emotional control.

Longitudinal findings remain limited but suggest that repeated immersive exposure over several weeks may produce sustained modulation of functional connectivity between prefrontal and limbic regions (Myers et al., 2025). Importantly, individual differences moderate these effects: baseline anxiety severity, trait mindfulness, and attentional control capacities influence the magnitude of regulatory engagement. Establishing causal mediation remains challenging, as many studies lack formal mediation analyses linking neural modulation to clinical symptom change. Expectancy effects, demand characteristics, and uncontrolled therapeutic components further complicate attribution of regulatory shifts specifically to immersive artistic features (Kim et al., 2023).

Despite these limitations, converging evidence indicates that immersive artistic engagement can transiently enhance top-down emotional regulation processes, particularly when tasks incorporate structured reflection and cognitive framing elements.

### 4.3 Attention networks and rumination disruption

A central neurocognitive hypothesis is that immersive artistic engagement may disrupt maladaptive rumination through modulation of attentional networks. Functional connectivity analyses have shown recruitment of the dorsal attention network (DAN) and frontoparietal control system during immersive tasks, accompanied by suppression of default mode network (DMN) activity associated with self-referential processing (Chen et al., 2025; Azarias et al., 2025; Catalogna et al., 2025b). Given that DMN hyperconnectivity is implicated in depressive rumination, its attenuation during immersive engagement provides a plausible neural mechanism for symptom reduction.

Behavioral assessments corroborate neural findings. Continuous performance tasks and dual-task paradigms reveal improved sustained attention, enhanced task-switching efficiency, and reduced cognitive interference following immersive exposure (Sokołowski et al., 2022). These attentional gains parallel those observed in mindfulness-based interventions, which similarly reduce DMN dominance and promote present-focused awareness (Wang P. et al., 2025; Misaki et al., 2023). However, immersive artistic engagement differs mechanistically by leveraging multisensory stimulation and interactive creativity, potentially amplifying attentional absorption beyond contemplative paradigms.

Correlational analyses indicate that reductions in DMN connectivity during immersive sessions are associated with decreases in repetitive negative thinking, supporting the proposition that attentional redeployment mediates rumination disruption (Lee et al., 2024). Although observed cognitive effects are generally modest, repeated sessions appear to consolidate attentional recalibration, yielding incremental improvements in executive control metrics (Drigas and Sideraki, 2024). Nonetheless, variability in task design, immersion intensity, and assessment timing limits cross-study comparability. Future investigations should employ standardized attentional measures and preregistered analytic strategies to strengthen causal inference.

### 4.4 Social brain networks in avatar interaction

Immersive environments incorporating avatar-mediated interaction have been shown to engage neural circuits central to social cognition. Functional imaging studies demonstrate activation of the temporoparietal junction, medial prefrontal cortex, and posterior superior temporal sulcus during embodied avatar interaction, reflecting engagement of mentalizing and perspective-taking processes (Milasi et al., 2025; Jimenez-Barragan et al., 2025). Compared with passive video-based communication, immersive avatar embodiment produces stronger activation within these networks, consistent with heightened social presence and experiential realism (Kiper et al., 2022; Arias-Álvarez et al., 2026).

Social affiliation and reward-related regions—including the ventral striatum and orbitofrontal cortex—are additionally recruited during collaborative artistic creation, particularly when content carries personal or cultural significance (Pan et al., 2025). This integration of social and reward circuitry suggests that immersive co-creation may simultaneously enhance belongingness and motivational salience (Figure 2). Individuals with social anxiety often exhibit attenuated baseline responses within social cognitive networks; however, anonymity and controlled self-presentation within avatar-mediated environments can partially normalize neural activation patterns by reducing perceived evaluative threat (Babu and Joseph, 2025).

Interpretation remains constrained by heterogeneity in task paradigms, limited sample sizes, and difficulty isolating the specific contribution of immersive embodiment from gaming or novelty effects. Nevertheless, converging evidence supports the premise that immersive social interaction enhances neural processes underlying empathy, affiliation, and social learning.

### 4.5 Neuroplasticity hypotheses and open questions

Evidence supporting durable neuroplastic changes induced by immersive artistic engagement is still emerging and limited by small samples and short follow-up intervals. Several longitudinal studies report functional connectivity alterations within prefrontal–limbic and attentional networks following repeated exposure, particularly when immersive sessions are integrated with structured reflection or therapeutic guidance (Mao et al., 2024; Juszko et al., 2023). Engagement parameters appear critical: moderate, repeated sessions (e.g., 30–45 min, three to five times per week) over multiple weeks are associated with measurable changes in BOLD coherence and network synchronization (Maslova et al., 2025).

Structural plasticity findings are less consistent. Reports of gray matter volume changes or cortical thickness modulation are limited by small sample sizes, short follow-up intervals, and absence of active control groups, precluding definitive conclusions (Kober et al., 2025). At present, claims of enduring structural reorganization remain speculative. Similarly, there is no robust evidence demonstrating molecular or epigenetic modulation resulting directly from immersive digital engagement. Analogies drawn from research on music therapy, meditation, or cognitive training provide suggestive parallels but cannot be directly extrapolated without empirical validation (Gholizadeh HamlAbadi et al., 2024; Noda and Noda, 2025).

Ethical and scientific prudence therefore require conservative interpretation, emphasizing functional adaptation within established neural circuits rather than unverified biological transformation. Future research should integrate multimodal assessment—combining behavioral indices, physiological measures, and neuroimaging—within preregistered, adequately powered designs. Large-scale collaborative consortia and standardized protocols would enhance replicability and strengthen causal inference (Han et al., 2024; Pillay et al., 2024). Translational challenges persist, particularly in adapting laboratory-based findings to real-world clinical contexts where immersion intensity, individual variability, and environmental factors must be carefully calibrated. The principal neurocognitive mechanisms through which immersive artistic engagement may exert therapeutic effects, together with the current level of supporting evidence and major methodological constraints, are synthesized in Table 2.

**Table 2:** Neurocognitive mechanisms of immersive artistic engagement: proposed processes, neural substrates, and strength of evidence.

**Mechanism / Neurocognitive process**	**Key brain regions / Networks involved**	**Main findings / Effects**	**Clinical relevance**	**Level of evidence and limitations**	**Reference(s)**
Reward and motivational enhancement	Ventral striatum, nucleus accumbens, VTA, mesolimbic pathways	Increased BOLD activation during active VR artistic engagement; enhanced with agency	Potential normalization of blunted reward in depression; reduced anhedonia	Moderate (fMRI/PET); small N, novelty confounds, indirect DA measures	Monaco et al. (2026), Singer et al. (2023), Tang et al. (2022), Lenormand and Piolino (2022), Van Timmeren et al. (2023), Ren et al. (2024), Dehghani et al. (2023), Cybinski et al. (2024), Int-Veen et al. (2025), and Goodpaster et al. (2026)
Prefrontal–limbic modulation and emotional regulation	dlPFC, vlPFC, amygdala, insula	Increased PFC activation; reduced amygdala reactivity; improved HRV and autonomic flexibility	Enhanced top-down emotion regulation; anxiety reduction	Moderate (fMRI + psychophysiology); limited longitudinal data, expectancy effects	Tang et al. (2022), Mugnol-Ugarte et al. (2024), Wang L. et al. (2025), Jeanne et al. (2025), Catalogna et al. (2025a), Myers et al. (2025), and Kim et al. (2023)
Attentional redeployment and rumination disruption	Dorsal attention network (DAN), frontoparietal control, DMN suppression	DAN/frontoparietal recruitment; DMN attenuation; improved sustained attention and task-switching	Reduced repetitive negative thinking and rumination	Moderate (fcMRI + behavioral); modest effects, task heterogeneity	Chen et al. (2025), Lee et al. (2024), Azarias et al. (2025), Catalogna et al. (2025b), Sokołowski et al. (2022), Wang P. et al. (2025), Misaki et al. (2023), and Drigas and Sideraki (2024)
Social cognition and affiliation via avatar interaction	TPJ, mPFC, pSTS, ventral striatum, OFC	Stronger activation in mentalizing networks; enhanced social presence and reward during co-creation	Improved belongingness; partial normalization in social anxiety	Moderate–preliminary; paradigm variability, embodiment vs. novelty confounds	Milasi et al. (2025), Jimenez-Barragan et al. (2025), Kiper et al. (2022), Arias-Álvarez et al. (2026), Pan et al. (2025), and Babu and Joseph (2025)
Functional neuroplasticity / connectivity changes	Prefrontal–limbic, attentional networks	Sustained changes in functional connectivity after repeated sessions (weeks)	Potential consolidation of regulatory and attentional gains	Preliminary (longitudinal fcMRI); small N, no active controls	Myers et al. (2025), Mao et al. (2024), Juszko et al. (2023), and Maslova et al. (2025)
Structural neuroplasticity	Cortical gray matter, thickness	Inconsistent / limited reports of volume or thickness changes	Not yet established	Weak / speculative; small samples, short follow-up, lacking controls	Kober et al. (2025)

In summary, immersive artistic engagement activates neurocognitive systems involved in reward, emotion regulation, attention, and social processing. Ventral striatal activity supports motivational salience, prefrontal–limbic interactions facilitate emotional regulation, attentional network recruitment reduces rumination, and avatar-based interaction strengthens social cognition and belonging. Although evidence for functional neuroplastic changes is emerging, interpretations should remain limited to observed alterations in connectivity and activation.

Taken together, These neurocognitive findings suggest that immersive artistic engagement may influence interacting systems involved in reward, attention, emotion regulation, and social cognition. The following section integrates these mechanisms within transdiagnostic models of psychopathology, focusing on processes relevant to depression and anxiety.

## 5 Transdiagnostic mechanisms relevant to depression and anxiety

**Figure 3:**
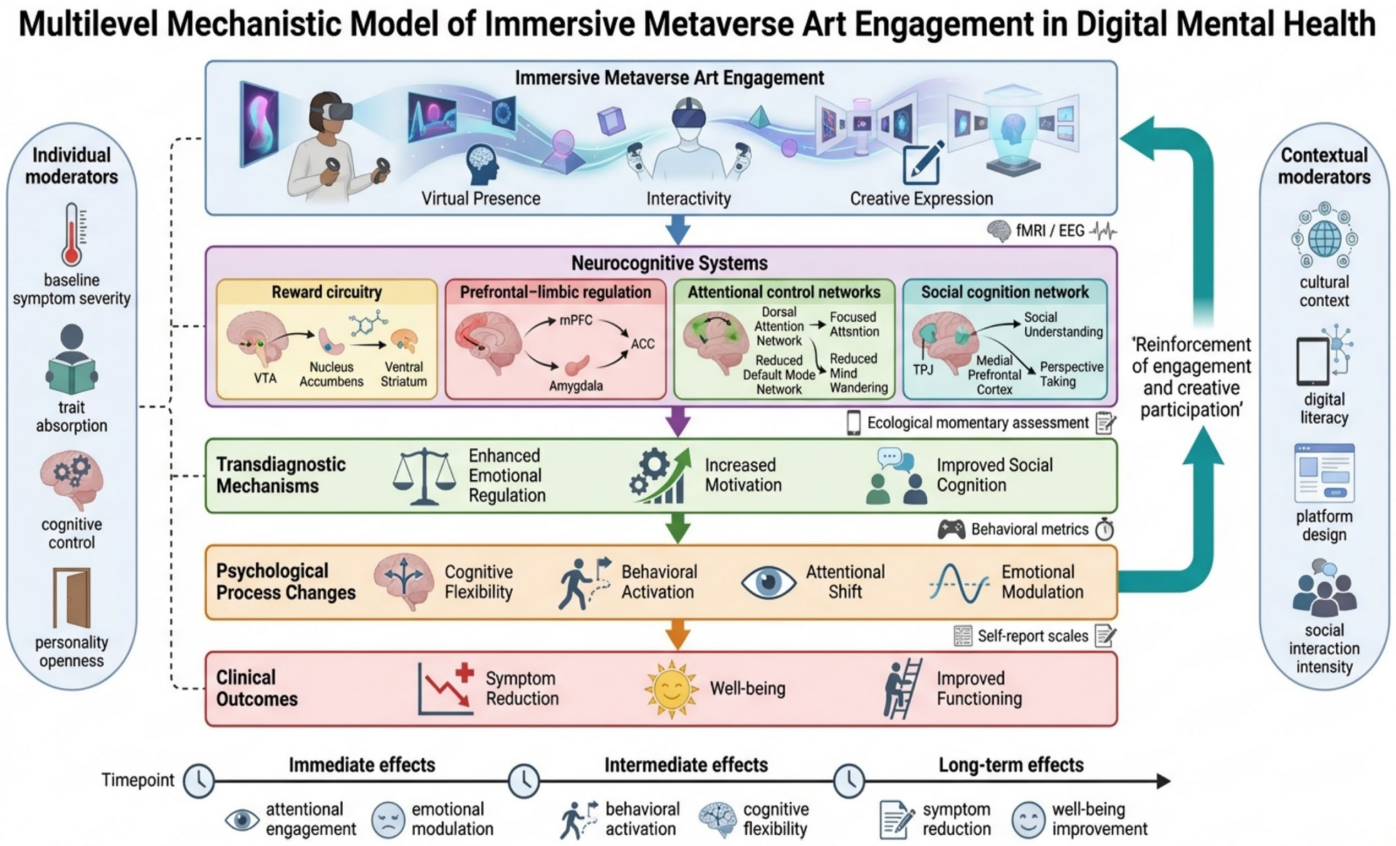
Multilevel mechanistic model of immersive metaverse art engagement in digital mental health. The model illustrates how multisensory creative and social engagement within immersive virtual environments may influence mental health through interacting neurocognitive systems and transdiagnostic psychological mechanisms. Engagement is proposed to modulate reward circuitry, prefrontal–limbic regulatory pathways, attentional control networks, and social cognition systems, which in turn affect core mechanisms such as rumination, experiential avoidance, anhedonia, and social isolation. These processes contribute to downstream changes in cognitive flexibility, behavioral activation, reward sensitivity, and social belonging, ultimately supporting reductions in depressive and anxiety symptoms and improvements in emotional well-being. Individual and contextual moderators, as well as temporal dynamics and reinforcing feedback loops, may shape these pathways.

As illustrated in Figure 3, immersive metaverse art engagement has been proposed to influence mental health through a cascade of transdiagnostic mechanisms that reshape core psychological processes and ultimately contribute to improvements in depressive and anxiety symptoms. Rather than addressing diagnostic categories in isolation, immersive metaverse art engages shared cognitive, affective, and behavioral processes that maintain symptomatology across disorders. By combining multisensory immersion, creative agency, and avatar-mediated interaction, these environments offer convergent pathways for symptom modulation. This section synthesizes evidence that emphasizing mechanistic specificity, moderators of response, and translational implications.

### 5.1 Rumination and cognitive inflexibility

Rumination—defined as repetitive, self-focused negative thinking—constitutes a central vulnerability and maintenance factor across depressive and anxiety disorders (Jingili et al., 2023; Lee et al., 2024). Contemporary immersive interventions assess rumination using validated instruments such as the Ruminative Response Scale (RRS) and the Perseverative Thinking Questionnaire (PTQ), enabling differentiation between state-level reductions and trait-level change (Ehring et al., 2011; Parola et al., 2017). Cognitive inflexibility, commonly evaluated via set-shifting paradigms and Wisconsin Card Sorting Test analogues, further sustains maladaptive cognitive loops by limiting adaptive problem-solving and perspective shifting (Doulou et al., 2025; Monni et al., 2023).

Immersive artistic engagement has been associated with disruption of internally oriented self-referential processing by reallocating attentional resources toward externally structured, multisensory tasks (Ma et al., 2025). Eye-tracking metrics, task-switching paradigms, and neuroimaging indices demonstrate measurable shifts from default-mode dominance to executive control network recruitment following immersive exposure (Lee et al., 2024; Gaddum et al., 2025). These attentional transitions correspond with improvements in executive flexibility and reduced perseverative cognition. Comparative studies suggest that immersive platforms produce reductions in repetitive negative thinking comparable to, and in some cases exceeding, mindfulness-based interventions, potentially due to the integration of creative agency, sensorimotor feedback, and goal-directed engagement (Cheng et al., 2025).

Importantly, Mediation analyses in preliminary studies indicate that decreases in rumination may partially account for a substantial proportion of observed reductions in depressive and anxiety symptoms, supporting a mechanism-specific effect rather than nonspecific mood enhancement (Li and Tang, 2024). Responsiveness varies as a function of baseline symptom severity, trait absorption, and cognitive control capacity. Repeated immersive sessions appear to consolidate gains in cognitive flexibility, although long-term durability remains under investigation (Wilczyńska et al., 2024; Esteller-Collado et al., 2025).

### 5.2 Avoidance and behavioral withdrawal

Experiential avoidance—characterized by efforts to evade distressing thoughts, emotions, or situations—represents a transdiagnostic maintenance process across affective disorders (Van Den Hout et al., 2011). Behavioral manifestations include diminished approach behavior, reduced goal-directed activity, and progressive social withdrawal (Seuling et al., 2024). Immersive artistic environments provide a structured context for graded exposure by embedding emotionally salient challenges within symbolic and controllable virtual settings (Cheng et al., 2025; Yang B. et al., 2024).

Design features such as adjustable task difficulty, guided exploration, and interactive narrative arcs enhance perceived psychological safety while preserving therapeutic challenge (Binder et al., 2022). Preliminary.

empirical findings indicate that immersive participation may reduce avoidance behavior, increases exploratory engagement, and promotes re-engagement with previously avoided stimuli both within virtual contexts and in daily life (Barrios et al., 2021; Shahid et al., 2024). These behavioral shifts align with process-based models of exposure and behavioral activation. Mediational analyses further demonstrate that reductions in experiential avoidance partially explain improvements in depressive and anxiety symptoms (Cheng et al., 2025).

However, boundary conditions warrant careful consideration. Unstructured or excessive immersion may inadvertently reinforce avoidance of offline stressors by providing an alternative escape context (Rizzo, 2018). When immersive engagement substitutes rather than supplements real-world coping, symptom improvement may be attenuated or transient. Accordingly, structured scaffolding, integration with offline behavioral goals, and reflective debriefing are essential to ensure that exposure remains adaptive rather than compensatory (Jingili et al., 2023).

### 5.3 Anhedonia and motivational deficits

Anhedonia—encompassing impairments in anticipatory and consummatory pleasure—contributes to diminished motivation and reduced goal-directed behavior across depressive and anxiety disorders (Kramer Freher et al., 2025). Laboratory paradigms such as effort-based decision-making tasks and reward responsiveness measures operationalize deficits in approach motivation and reward sensitivity (Lu et al., 2025b; Chen K. et al., 2021).

Immersive artistic participation directly engages motivational circuitry through interactive creativity, real-time feedback, and visible progress toward self-defined goals (Chen K. et al., 2021). Co-creative tasks, particularly when embedded within collaborative or socially reinforced environments, enhance anticipatory reward signaling and sustain persistence (Chen K. et al., 2021; Sun et al., 2026). Neurobehavioral evidence, including striatal BOLD activation and autonomic indices of engagement, suggests that immersive exposure increases reward responsiveness in a manner comparable to traditional behavioral activation protocols (Kramer Freher et al., 2025).

Longitudinal data indicate that repeated immersive engagement may generalize to offline functioning, increasing daily activity levels, persistence in goal pursuit, and willingness to initiate effortful tasks (Lu et al., 2025b; Sun et al., 2026). Nonetheless, variability in response underscores the importance of personalization. Baseline anhedonia severity, prior creative experience, and trait motivational orientation moderate the magnitude of benefit (Chen K. et al., 2021). Tailored task calibration and autonomy-supportive design may therefore optimize motivational gains and reduce attrition.

### 5.4 Social isolation and belongingness deficits

Perceived social isolation—defined as the subjective experience of disconnection from meaningful relationships—represents a robust predictor of depressive and anxiety symptom severity (Kenyon et al., 2023). Immersive avatar-mediated environments create opportunities for social affiliation, enhancing perceived co-presence, group cohesion, and interpersonal closeness (Gleich and Schöbel, 2026; Derda et al., 2025). Subjective connectedness is commonly assessed using instruments such as the Social Connectedness Scale and adapted loneliness measures within VR contexts (Kenyon et al., 2023).

Avatar embodiment appears to enhance empathic processing and willingness to engage in collaborative tasks, even among individuals with elevated social anxiety (Angerbauer et al., 2025). Shared artistic creation, synchronized interaction, and culturally resonant narratives foster psychological safety and enable rehearsal of social skills within low-risk settings (Angerbauer et al., 2025; Li et al., 2024). Emerging longitudinal findings suggest that immersive social affiliation predicts improvements in offline social functioning, mediated by increased perceived safety, strengthened identity alignment, and positive interpersonal feedback loops (Kenyon et al., 2023; Derda et al., 2025).

Moderating variables include baseline social anxiety, digital literacy, and cultural norms surrounding self-presentation and collectivism. Additionally, excessive reliance on virtual relationships without integration into offline networks may increase digital relational dependency if not appropriately scaffolded (Gleich and Schöbel, 2026; Salehi et al., 2025). Structured integration strategies are therefore necessary to translate immersive social gains into sustainable real-world connectedness.

In aggregate, immersive artistic interventions target four interrelated transdiagnostic mechanisms—rumination, avoidance, anhedonia, and social disconnection—through integrated cognitive, affective, and behavioral pathways. By interrupting repetitive negative thinking, facilitating graded exposure, enhancing motivational salience, and promoting avatar-mediated social engagement, immersive platforms offer a process-oriented approach to depression and anxiety management. Although current evidence is encouraging, definitive conclusions require larger randomized trials, standardized mechanistic measures, and multimodal longitudinal designs capable of clarifying causal pathways and identifying moderators of responsiveness.

By conceptualizing immersive metaverse art through shared transdiagnostic mechanisms, the preceding framework provides an integrative structure linking engagement-related processes, identity-related experiences, and neurocognitive modulation to psychological outcomes. The following section builds upon this framework by examining disorder-specific evidence in depression and anxiety populations, thereby evaluating how these interconnected mechanisms may manifest across different clinical contexts.

## 6 Disorder-specific evidence

Immersive artistic interventions have been increasingly evaluated for disorder-specific efficacy in depression and anxiety. Empirical studies (primarily small-to-moderate RCTs and pilot trials) provide insight into acute affective modulation, behavioral activation, exposure-based mechanisms, and transdiagnostic effects across clinical and subclinical populations. This section synthesizes contemporary findings, emphasizing effect magnitude, mediational pathways, moderators of response, and methodological constraints. Table 3 provides a structured summary of the principal empirical studies — including RCTs, pilot trials, feasibility investigations, and meta-analyses — evaluating immersive VR-based artistic and therapeutic interventions for depression, anxiety, and their comorbidity.

**Table 3:** Summary of empirical studies on immersive VR interventions for depression and anxiety.

**Study focus / Population**	**Intervention type**	**Key outcomes / Effect Sizes**	**Mediators / Mechanisms**	**Moderators / Notable findings**	**Main limitations**	**Reference(s)**
Depression (general and subclinical)	Immersive VR artistic/creative tasks	Acute mood improvement (*d* ≈ 0.45–0.85); sustained with repetition	Behavioral activation, intrinsic motivation, perceived agency	Age, baseline severity, digital literacy; higher effects in moderate–severe cases	Small samples, short follow-up, novelty effects	Paul et al. (2024), Lem et al. (2024), Jimenez-Barragan et al. (2025), Kramer Freher et al. (2025), Lu et al. (2025b), Cortés-Pérez et al. (2025), Ke et al. (2025), Liu W. et al. (2025), Wang S. et al. (2025), Kukharuk et al. (2025), Kim B. et al. (2025), Pratviel et al. (2024), Jespersen et al. (2024), and Reategui-Rivera et al. (2024)
Depression – RCT with active controls	XR-enhanced behavioral activation	Superior reduction in depressive affect vs. passive digital; higher engagement	Approach behavior, creative agency	User choice and customization predict adherence	Attrition 10–25%, lack of long-term data	Paul et al. (2024), Kim B. et al. (2025), and Robinson et al. (2025)
Depression – adjunctive / treatment-resistant	Immersive protocols + pharmacotherapy/psychotherapy	Modest additive mood benefits	Motivational reinforcement	Limited evidence in resistant cases	Pilot data only, small N	Acuña-Rodríguez et al. (2025) and Zeng et al. (2025)
Anxiety – social & performance anxiety	VR-based graded exposure + avatar mediation	Reduced avoidance, state anxiety (*d* ≈ 0.35–0.70); comparable to *in vivo*	Fear extinction, perceived control, anonymity	Greater agency → larger arousal reduction; clinician guidance enhances effects	Risk of reinforcing avoidance if unsupervised	Zeng et al. (2025), Derda et al. (2025), Tan et al. (2025), Uduwa Vidanalage et al. (2025), Chard et al. (2023), and Mulvaney et al. (2026)
Anxiety – social contexts (incl. Stuttering, immigrants)	Avatar-mediated interaction and embodiment	Improved social confidence, reduced anticipatory anxiety	Social presence, reduced evaluative threat	Anonymity aids initiation; generalization requires guided transfer	Limited long-term follow-up	Pan et al. (2025), Chard et al. (2023), Holt et al. (2025), Chen V. H. H. et al. (2021), Rus-Calafell et al. (2025), and Takemoto et al. (2025)
Mixed depression + anxiety / comorbid	Immersive VR meditation, relaxation, or remediation	Transdiagnostic gains in mood, rumination, avoidance	Reduced rumination, increased approach behavior	Comorbidity complexity, executive control	Heterogeneity in protocols, small–moderate N	Jingili et al. (2023), Lee et al. (2024), Jimenez-Barragan et al. (2025), Hubbard et al. (2025), and Primavera et al. (2024)
Physiological correlates (both disorders)	Immersive exposure / relaxation environments	Increased HRV associated with anxiety/stress reduction	Autonomic regulation (vagal tone)	Preliminary; small samples, measurement variability	Short follow-up, protocol heterogeneity	Pratviel et al. (2024), Vann-Adibe et al. (2025), Benvegnù et al. (2026), and Nath et al. (2024)

### 6.1 Depression-focused findings

RCTs evaluating immersive virtual reality (VR) and interactive artistic platforms for depression have reported immediate improvements in self-reported mood following single-session or brief multi-session exposure (Jimenez-Barragan et al., 2025; Liu W. et al., 2025). Frequently employed outcome measures include the Beck Depression Inventory-II (BDI-II), Patient Health Questionnaire-9 (PHQ-9), and Visual Analogue Mood Scales (VAMS) (Cortés-Pérez et al., 2025; Ke et al., 2025). Reported effect sizes for immediate post-session mood enhancement range from moderate to large (Cohen’s *d* ≈ 0.45–0.85), depending on intervention intensity, session frequency, and participant characteristics (Wang S. et al., 2025). Acute benefits typically persist for several hours to several days, with sustained improvement contingent upon repeated engagement and integration into structured activity routines (Kukharuk et al., 2025).

Comparative analyses suggest that immersive art–based interventions yield short-term effects comparable to behavioral activation and, in some cases, cognitive behavioral therapy (CBT), particularly in enhancing positive affect and experiential engagement (Robinson et al., 2025). Approximately 40–50% of contemporary trials incorporate active control conditions—such as non-immersive digital activities or structured psychoeducation—to isolate immersive-specific effects from novelty or expectancy influences (Paul et al., 2024). Across these comparisons, immersive platforms generally demonstrate superior reductions in depressive affect and higher engagement indices relative to passive digital media, likely attributable to multisensory stimulation, user agency, and attentional absorption (Kramer Freher et al., 2025).

Behavioral activation emerges as a central mediator of symptom change. Immersive creative participation increases goal-directed behavior by embedding tangible, self-directed tasks within structured environments, reinforcing approach tendencies and intrinsic motivation (Kim B. et al., 2025). Behavioral metrics—including task completion rates, in-platform activity logs, and validated approach behavior scales—correlate with depressive symptom reduction (Lu et al., 2025b). Several mediation analyses indicate that increases in intrinsic motivation and perceived creative agency statistically account for a significant portion of observed symptom improvement (Pratviel et al., 2024). User agency—operationalized as choice of activity, avatar customization, and narrative control—predicts sustained engagement and cumulative gains across sessions (Vann-Adibe et al., 2025). Process-based analyses further confirm that immersive participation promotes approach-oriented behavior, which contributes to both immediate mood enhancement and incremental improvement over time (Lem et al., 2024).

Moderators of response include age, baseline symptom severity, and contextual variables. Adolescents demonstrate meaningful affect regulation and behavioral activation benefits; however, cognitive control limitations and attentional variability necessitate age-sensitive design parameters (Jespersen et al., 2024). Older adults experience comparable short-term mood improvements, though cognitive load, interface complexity, and usability factors influence adherence and effect magnitude (Reategui-Rivera et al., 2024). Baseline severity appears to moderate responsiveness, with individuals exhibiting moderate-to-severe depressive symptoms often demonstrating more pronounced improvements under high-engagement protocols (Tan et al., 2025). Evidence in treatment-resistant depression remains limited; pilot studies suggest modest additive mood benefits when immersive interventions are integrated adjunctively with pharmacotherapy or psychotherapy (Cheng et al., 2025).

Digital literacy and socioeconomic context also influence accessibility and adherence. Familiarity with interactive technologies predicts initial engagement, whereas lower digital competence and higher baseline symptom burden are associated with increased dropout risk (Zeng et al., 2025). Reported attrition rates range from approximately 10–25%, with predictors including perceived task difficulty and motivational deficits (Uduwa Vidanalage et al., 2025). While acceptable relative to other digital interventions, these rates underscore the importance of adaptive scaffolding and user-centered design.

### 6.2 Anxiety-focused findings

Immersive artistic environments function as structured graded exposure contexts for diverse anxiety presentations by embedding anxiety-relevant stimuli within controllable symbolic frameworks (Chard et al., 2023). RCTs demonstrate that VR-based exposure to social, performance, or situational stressors reduces avoidance behaviors and facilitates fear extinction processes (Mulvaney et al., 2026). Perceived control within immersive contexts significantly moderates outcomes: greater user agency is associated with larger reductions in physiological arousal and subjective anxiety ratings (Chen V. H. H. et al., 2021).

Anxiety outcomes are typically measured using state–trait anxiety inventories, behavioral approach tasks, and avoidance frequency logs (Holt et al., 2025). Comparative trials indicate that immersive exposure achieves reductions in avoidance comparable to traditional *in vivo* exposure, while often enhancing participant comfort, perceived safety, and adherence (Takemoto et al., 2025). Clinician-guided immersive protocols generally yield stronger effects than fully self-directed formats; however, structured self-paced interventions also produce measurable symptom reduction when adequately scaffolded (Rus-Calafell et al., 2025).

Avatar-mediated anonymity represents a distinct advantage for socially anxious populations. Anonymity reduces perceived evaluative threat, lowers anticipatory anxiety, and increases willingness to initiate social interaction within immersive group contexts (Derda et al., 2025). Empirical findings demonstrate that both fully anonymous and partially identity-linked environments can improve short-term social confidence; however, complete detachment from offline identity may limit generalization if not accompanied by guided integration strategies (Jingili et al., 2023; Pan et al., 2025). Longitudinal assessments suggest that immersive rehearsal contributes to reductions in real-world social anxiety symptoms, mediated by repeated exposure, perceived safety, and enhanced agency (Hubbard et al., 2025).

Potential risks must also be considered. Excessive reliance on avatar-mediated socialization may inadvertently reinforce avoidance of offline interpersonal challenges, particularly when immersive engagement is unsupervised or substitutes for real-world exposure (Reategui-Rivera et al., 2024). Accordingly, integration with graduated real-world behavioral goals is critical to maximize transfer.

Physiological correlates of anxiety reduction, particularly heart rate variability (HRV), have been examined as objective indices of autonomic regulation (Pratviel et al., 2024). Preliminary findings indicate that HRV increases during immersive exposure are associated with reduced subjective anxiety; however, these studies are limited by small sample sizes, measurement heterogeneity, and short follow-up periods (Vann-Adibe et al., 2025; Benvegnù et al., 2026). Standardization of HRV acquisition protocols and longitudinal monitoring is necessary to clarify whether autonomic modulation persists beyond acute sessions (Nath et al., 2024).

### 6.3 Comorbidity and overlapping pathways

Given the high prevalence of comorbid depressive and anxiety symptoms, many immersive intervention trials enroll mixed-diagnostic samples (Primavera et al., 2024). Such interventions often target shared transdiagnostic processes—rumination, avoidance, motivational deficits, and social withdrawal—yielding improvements across both depressive and anxiety measures (Lee et al., 2024). Mediation analyses indicate that reductions in maladaptive rumination and increases in approach behavior partially account for symptom improvement across diagnostic domains (Paul et al., 2024).

Differential responsiveness patterns have been observed. Depressive symptoms tend to show larger improvements in affective and motivational dimensions, whereas anxiety symptoms respond robustly to exposure-based and social engagement mechanisms (Kramer Freher et al., 2025). Baseline severity, comorbidity complexity, and executive control capacity moderate these differential effects. Importantly, behavioral activation and graded exposure mechanisms appear synergistic in comorbid populations, jointly addressing overlapping vulnerabilities (Kim B. et al., 2025).

Evidence for immersive interventions in clinically diagnosed major depressive disorder with comorbid anxiety remains limited but encouraging. Several studies evaluate immersive protocols adjunctive to pharmacotherapy, reporting additive symptom reduction without significant adverse effects (Robinson et al., 2025). Safety monitoring typically includes clinician oversight, continuous distress ratings, and structured debriefing to mitigate potential psychological risks (Jimenez-Barragan et al., 2025).

From a methodological perspective, disorder-specific research is dominated by RCTs, pilot trials, and feasibility studies with sample sizes ranging from approximately 20–120 participants (Lu et al., 2025b). Follow-up durations rarely extend beyond one month, limiting inference regarding sustained remission. Reported effect sizes for depressive symptom reduction are generally moderate (*d* ≈ 0.45–0.85), with slightly lower but still clinically meaningful effects for anxiety outcomes (*d* ≈ 0.35–0.70) (Tan et al., 2025). Substantial heterogeneity persists in session duration, frequency, platform architecture, and therapeutic scaffolding, complicating direct cross-study comparison (Cheng et al., 2025). Risk-of-bias assessments highlight concerns related to small samples, lack of blinding, and potential publication bias, although preregistration and transparent reporting practices are increasingly adopted (Zeng et al., 2025; Kukharuk et al., 2025). Replication studies remain scarce, underscoring the need for standardized protocols and multicenter collaboration to consolidate the evidence base (Uduwa Vidanalage et al., 2025).

While disorder-specific findings provide preliminary support for immersive artistic interventions, interpretation of these outcomes requires broader contextualization within existing therapeutic paradigms and critical methodological considerations. The following section therefore situates immersive metaverse art in relation to traditional art therapy and related interventions, while also addressing conceptual limitations, novelty effects, accessibility concerns, and potential psychological risks that may influence clinical applicability.

## 7 Comparative and critical perspectives

The expansion of immersive metaverse-based artistic interventions necessitates systematic comparison with established expressive therapies and careful examination of methodological and ethical constraints. Although preliminary findings indicate therapeutic potential, critical scrutiny is required to delineate clinical boundaries, prevent overinterpretation of early results, and clarify whether immersive art confers mechanistic advantages beyond existing modalities.

### 7.1 Metaverse art versus traditional art therapy

Traditional art therapy is grounded in psychodynamic, humanistic, and cognitive-behavioral traditions, emphasizing symbolic expression, affect externalization, and meaning-making through tactile artistic production (Quinn et al., 2025; Joschko et al., 2024). Symbolically, artworks function as transitional objects (Winnicott) and vehicles for projection and containment of unconscious material, enabling clients to externalize internal conflicts in metaphorical form, bypass verbal defenses, and achieve gradual psychic integration (McCullough, 2009). Clinically, this mechanism is especially potent for individuals with depression and anxiety who experience alexithymia, rumination, or interpersonal avoidance, as it provides a non-verbal, low-threat pathway to insight and emotional processing that traditional talk therapy may not readily access (Hadjipanayi et al., 2023; Joschko et al., 2024). Creative output is conceptualized as a transitional object that enables projection, containment, and integration of emotional material (Hadjipanayi et al., 2023). Material engagement constitutes a core embodied mechanism in traditional art therapy. The physical properties of art media—resistance of clay, fluidity of paint, texture of collage—generate immediate sensorimotor and proprioceptive feedback loops that ground abstract emotional states in concrete bodily experience, promote physiological down-regulation, and foster a tangible sense of mastery and transformation (McCullough, 2009; Haeyen, 2024). This haptic interaction is clinically nuanced in its capacity to address somatic symptoms, dissociation, and depressive withdrawal, offering neuroceptive safety and embodied catharsis that complement cognitive-symbolic work (Haeyen, 2024). Emotional regulation emerges through sensorimotor engagement, therapist-guided reflection, and relational attunement within a structured therapeutic alliance (Richesin et al., 2021). The therapeutic alliance is not merely supportive but actively therapeutic: the therapist’s empathetic witnessing, interpretive scaffolding, and co-construction of meaning around the artwork create a secure relational container, facilitate transference exploration (including transference to the artwork itself), and enable real-time emotional containment and interpersonal repair (Hilbuch et al., 2016).

Immersive metaverse art overlaps with these principles through digital symbolic manipulation, creative agency, avatar embodiment, and interactive narrative construction, thereby facilitating cognitive reappraisal, emotional processing, and identity exploration (Paul et al., 2024). However, digital symbolism tends to be more explicit, iterative, and customizable (e.g., real-time avatar or environment modification), which can accelerate self-expression and narrative flexibility while potentially reducing the depth of spontaneous, unconscious projection afforded by the ambiguity and physicality of traditional materials. However, the modalities differ substantially in embodied engagement. In-person art therapy involves haptic feedback, material resistance, and fine motor coordination, processes theorized to strengthen somatosensory grounding and affect regulation (Paquin et al., 2023). Most immersive platforms, in contrast, emphasize visual–auditory immersion currently limited tactile/haptic integration (although emerging haptic technologies may narrow this gap). Available experimental comparisons suggest similar short-term mood improvements but differential somatosensory activation patterns across tactile and virtual contexts (Pal and Arpnikanondt, 2024).

Therapist involvement represents another core distinction. Traditional art therapy depends on co-constructed meaning and relational containment, with therapist responsiveness shaping interpretive depth and emotional integration (Buragohain et al., 2025). The live therapeutic alliance provides immediate containment for intense affect, real-time interpretation of symbolic content, and management of transference dynamics that are central to clinical change (Hilbuch et al., 2016). Immersive interventions range from fully self-guided systems to clinician-guided or hybrid models. Guided immersive formats tend to produce stronger outcomes, particularly among individuals with complex symptom profiles, whereas self-directed models enhance scalability (Seon et al., 2025). In immersive settings the alliance can be approximated through avatar-mediated or synchronous remote guidance, yet it may be attenuated in fully automated formats, potentially limiting opportunities for nuanced transference work and crisis containment compared with in-person relational attunement (Buragohain et al., 2025; Seon et al., 2025). Effect sizes reported for traditional art therapy in depression and anxiety (Hedges’ *g* ≈ 0.45–0.75) (Bojić et al., 2026) are broadly comparable to those observed in immersive artistic interventions (*d* ≈ 0.40–0.85) (Kim and Kim, 2023), though immersive studies generally include shorter follow-up periods and greater protocol heterogeneity.

Delivery structure further differentiates modalities. Traditional art therapy typically involves weekly 60–90-min sessions over 8–16 weeks, whereas immersive programs vary from single-session exposures to multi-week digital interventions (Selaskowski et al., 2024). Reported dropout rates are similar (10–25%), yet immersive adherence is more strongly moderated by digital literacy and hardware accessibility (Birckhead et al., 2019).

Scalability constitutes a principal advantage of immersive platforms. Conventional art therapy requires licensed practitioners and physical space, limiting geographic reach (Goulet et al., 2025). Immersive systems may partially alleviate provider shortages and rural access barriers, though dependence on hardware, broadband infrastructure, and technical maintenance introduces new inequities (Zhang et al., 2025). Preliminary cost modeling suggests potential reductions in per-user cost at scale, offset by substantial development expenditures (Ding et al., 2025). Ethical concerns arise when automated systems replace relational containment, particularly regarding crisis responsiveness and contextual interpretation of symbolic content. The principal similarities and differences between immersive metaverse-based artistic interventions and traditional art therapy, including therapeutic mechanisms, practical considerations, and evidence limitations, are summarized in Table 4.

**Table 4:** Comparison between immersive metaverse art therapy and traditional art therapy.

**Dimension**	**Traditional art therapy**	**Immersive metaverse art therapy**	**Key similarities / Overlaps**	**Key differences / Considerations**	**Reference(s)**
Therapeutic foundations	Psychodynamic, humanistic, CBT; symbolic expression as transitional phenomena for projection, containment, and integration of unconscious material; affect externalization and meaning-making	Symbolic manipulation, creative agency, cognitive reappraisal, emotional processing, dynamic, interactive, and customizable digital symbolism (avatars, virtual environments)	Reliance on symbolic/creative processes for affect regulation	Traditional emphasizes spontaneous unconscious projection via material ambiguity; immersive enables explicit, iterative, narrative-driven symbolism but may reduce analog depth	Paul et al. (2024), Hadjipanayi et al. (2023), Quinn et al. (2025), Joschko et al. (2024), and Richesin et al. (2021)
Embodied / Sensorimotor engagement	Haptic feedback, material resistance, fine motor coordination; somatosensory grounding and embodied catharsis via tactile manipulation	Primarily visual–auditory; limited tactile integration (emerging haptics may address this)	Facilitates affect transformation via creative output	Traditional provides stronger haptic/proprioceptive grounding and anti-dissociative effects; immersive offers safety, undoability, and scalability but less direct embodiment	Richesin et al. (2021), Paquin et al. (2023), and Pal and Arpnikanondt (2024)
Therapist involvement / Therapeutic alliance	Essential; co-constructed meaning, relational containment, interpretive guidance via live therapeutic alliance, transference work, and real-time emotional containment	Variable: self-guided to clinician-guided/hybrid (avatar-mediated or synchronous guidance can approximate alliance)	Guided formats enhance outcomes in complex cases	Traditional offers physical presence, nuanced transference interpretation, and immediate containment; immersive enhances scalability but risks dilution of relational depth and crisis response	Buragohain et al. (2025) and Seon et al. (2025)
Effect sizes (Depression/Anxiety)	Hedges’ *g* ≈ 0.45–0.75	Cohen’s *d* ≈ 0.40–0.85	Comparable short-term mood improvements	Immersive studies: shorter follow-up, higher protocol heterogeneity	Bojić et al. (2026) and Kim and Kim (2023)
Session structure / Duration	Weekly 60–90 min sessions, 8–16 weeks	Single-session to multi-week; highly variable	Similar dropout rates (10–25%)	Immersive more flexible but moderated by digital literacy/hardware access	Selaskowski et al. (2024) and Birckhead et al. (2019)
Scalability and accessibility	Limited by licensed practitioners, physical space	High potential; reduces geographic/provider barriers	Both aim to enhance access to creative expression	Immersive introduces hardware, broadband, cost inequities; development expenses high	Ding et al. (2025), Goulet et al. (2025), and Zhang et al. (2025)
Ethical / Practical concerns	Relational crisis response, symbolic interpretation	Risk of replacing containment; crisis handling in automated systems	Potential for emotional processing via art	Immersive: novelty effects, accessibility barriers, cultural incongruence risks	Buragohain et al. (2025), Paquin et al. (2023), and Goulet et al. (2025)

### 7.2 Novelty effects and placebo concerns

Technological novelty may transiently elevate mood and engagement independent of specific therapeutic mechanisms. Several studies address this by comparing high-immersion VR with low-immersion or familiar digital formats (Dwivedi et al., 2022). Engagement benefits sometimes diminish with repeated exposure, indicating partial novelty effects (Jingili et al., 2023). However, trials incorporating active control conditions—such as 2D digital art platforms, relaxation media, or non-interactive VR—provide stronger evidence that immersive affordances confer benefits beyond general expectancy (Loetscher et al., 2023). Expectancy is typically assessed using treatment credibility and pre-intervention expectation measures, although participant blinding is rarely feasible (Pedram and Piatkowski, 2025). Partial blinding of outcome assessors is reported in a minority of studies (Machado et al., 2025). Observed symptom reductions often exceed typical placebo response magnitudes in depression trials (d ≈ 0.30) (Szekely et al., 2025), yet limited statistical power constrains definitive interpretation. Multi-session protocols demonstrating maintained improvement beyond initial exposure strengthen the argument for mechanisms extending beyond novelty (Du Plessis and Jordaan, 2024). Nonetheless, rigorous active comparators and transparent expectancy reporting remain necessary to mitigate bias.

### 7.3 Risks: escapism, dependency, and identity diffusion

Immersive engagement may reinforce maladaptive avoidance if used to escape offline stressors. Research on problematic gaming identifies avoidance coping, diminished self-concept clarity, and baseline psychopathology as vulnerability factors for dependency-like patterns (Pal and Arpnikanondt, 2024). Behavioral dysregulation is operationalized through excessive use, impaired role functioning, and loss of control, often assessed via adapted gaming disorder scales (Wang Y. et al., 2025).

Differentiating therapeutic engagement from compulsive use requires evaluation of functional impairment, emotion regulation strategies, and alignment with real-world goals (Paquin et al., 2023). Although immersive artistic interventions are not consistently associated with harm, unsupervised excessive engagement may exacerbate avoidance behaviors (Mazumdar et al., 2024).

Avatar-mediated identity experimentation can support adaptive self-exploration and social rehearsal; however, prolonged detachment from offline identity may reduce self-concept coherence. Evidence linking immersive identity use to fragmentation remains limited, though reduced self-concept clarity has been observed among high-frequency virtual identity users (Buragohain et al., 2025). Adolescents and individuals with dissociative vulnerabilities may require particular caution (Wang Y. et al., 2025). Structured session limits and guided reflective integration may mitigate these risks.

### 7.4 Cultural bias, accessibility, and replicability

Immersive environments often reflect dominant cultural aesthetics, potentially limiting inclusivity (Chakrabarti, 2025). Culturally incongruent content can reduce engagement and perceived relevance (Buragohain et al., 2025), whereas adapted interventions incorporating localized narratives and language customization demonstrate improved satisfaction and emotional resonance (Haeussl et al., 2026). Socioeconomic inequities further constrain dissemination, as hardware cost and broadband requirements limit access (Cheng et al., 2025). Digital literacy moderates benefit and adherence (Baschab et al., 2026), and representation of rural or underserved populations in trials remains limited, despite emerging telehealth-integrated VR initiatives (Lu et al., 2025b).

Methodological heterogeneity compounds these concerns. Variability in immersion level, session duration, therapist involvement, and outcome measurement impedes cross-study comparability (Cheng et al., 2025). Sample sizes frequently range from 20 to 100 participants, rendering many studies underpowered for mediation analyses (Hubbard et al., 2025). Preregistration and reporting transparency have improved but remain inconsistent (Haeussl et al., 2026), and replication studies are scarce (Buragohain et al., 2025). Long-term follow-ups are infrequent, and mediator analyses typically rely on short-term statistical models (Lu et al., 2025b). Risk-of-bias assessments highlight concerns regarding selection bias, performance bias, and selective reporting (Cheng et al., 2025).

Beyond these methodological and contextual concerns, several broader limitations of the current evidence base should also be considered. Despite promising preliminary findings, several limitations should be acknowledged. First, this manuscript is a narrative and conceptual review rather than a systematic review. Although this approach was appropriate for an emerging and interdisciplinary field, the absence of a formal systematic search framework (e.g., PRISMA-guided selection and quantitative synthesis) increases the possibility of selection bias and interpretive subjectivity. Consequently, the proposed multilevel model should be interpreted as a hypothesis-generating framework rather than a definitive or universally representative account of immersive metaverse interventions.

Second, the current evidence base remains methodologically limited. Much of the literature consists of pilot studies and small-scale trials with short follow-up periods, heterogeneous intervention protocols, and inconsistent control conditions, limiting generalizability and predictive validity. In addition, some reported benefits may partially reflect novelty effects associated with immersive VR technology rather than stable therapeutic mechanisms. Neurocognitive findings should also be interpreted cautiously due to limited replication and modest sample sizes.

Strengthening the field will require adequately powered randomized controlled trials, active comparators, standardized outcome measures, preregistration, and extended follow-up. Multimodal assessment and independent replication will be essential to establish reliable mechanisms and ensure responsible clinical translation. As the literature matures, systematic reviews and meta-analyses will be necessary to validate the proposed pathways, estimate effect sizes, and identify moderators of responsiveness across diverse populations and clinical contexts.

Despite these methodological and ethical challenges, the convergence of engagement-related, identity-based, neurocognitive, and transdiagnostic findings suggests that immersive artistic interventions may hold translational potential when implemented within structured clinical frameworks. The following section therefore examines how these interconnected mechanisms may be integrated into hybrid therapeutic models, telepsychology systems, and scalable mental health interventions, with emphasis on feasibility, personalization, and clinical oversight.

## 8 Translational and clinical integration

Translating immersive metaverse-based artistic interventions into routine clinical and public health practice requires more than evidence of short-term efficacy. Building directly on the multilevel conceptual framework and transdiagnostic mechanisms, sustainable implementation demands explicit mapping of core technological affordances and psychological mediators—engagement (flow/presence), identity (avatar embodiment/cultural symbolism), and neurocognitive pathways—to concrete clinical protocols. This includes pathway-specific intervention examples, defined therapist roles, explicit patient selection criteria, standardized implementation structures, and proactive risk-management procedures for potential adverse effects such as overdependence and maladaptive avoidance. Sustainable implementation depends on structured integration with established psychotherapies, cautious validation of biomarker-informed personalization, scalable infrastructure, and rigorous ethical and regulatory oversight. Translational maturity therefore hinges on feasibility, safety, clinician training, and compatibility with existing healthcare delivery systems.

### 8.1 Hybrid integration with CBT and telepsychology

Empirical work increasingly positions immersive artistic environments as adjunctive components within cognitive behavioral therapy (CBT) frameworks (Zeng et al., 2025; Kumpulainen et al., 2024). In hybrid models, therapist-led telepsychology sessions are combined with structured immersive assignments completed between sessions. Patients may engage in symbolic exposure exercises, behavioral activation tasks, or creative re-authoring of distress narratives within immersive environments, followed by therapist-guided cognitive processing and schema integration (Ito et al., 2023). This model capitalizes on complementary mechanisms: immersive platforms amplify experiential salience and emotional engagement, while CBT provides structured cognitive reappraisal and consolidation (Lundin et al., 2023). Graded exposure conducted within controllable virtual contexts may enhance inhibitory learning processes central to contemporary exposure theory (Wu et al., 2021). In depression-focused hybrids, immersive creative tasks function as behavioral activation modules that increase approach behavior, subsequently reinforced during therapy sessions (Lu et al., 2025b).

Within stepped-care systems, immersive modules are increasingly conceptualized as low-intensity entry points in digital mental health ecosystems (Jingili et al., 2023). Patient selection criteria prioritize individuals with mild-to-moderate depressive or anxiety symptoms (PHQ-9/GAD-7 scores 10–19), adequate digital literacy (assessed via brief screening), motivation for creative or experiential engagement, and low baseline risk for cybersickness or dissociation (e.g., screened via trait absorption and dissociation proneness scales (McDonald et al., 2025)). Patients with severe psychopathology, history of severe dissociation, or limited technological access are directed to traditional care pathways.

Pathway-specific clinical examples further illustrate practical application. In the engagement pathway, patients exhibiting prominent rumination receive immersive artistic creation tasks calibrated to induce flow states (e.g., adaptive virtual painting or sculpting with real-time difficulty adjustment; Guerra-Tamez, 2023; Yang X. et al., 2024); therapists monitor in-session flow via brief self-report or physiological indices and debrief to reinforce attentional reorientation, linking virtual absorption to daily behavioral activation plans. In contrast, the identity pathway targets social avoidance and belonging deficits through avatar-mediated collaborative art sessions in culturally resonant virtual spaces; patients experiment with symbolic self-representation and co-creation, after which therapists facilitate reflective discussion of the Proteus effect and narrative reconstruction to promote offline social rehearsal and identity coherence (Fusaro et al., 2025; Lin et al., 2025).

Therapist roles are clearly delineated and essential for safe, effective delivery. Clinicians (a) select and customize immersive modules according to the patient’s dominant transdiagnostic mechanism and baseline profile, (b) provide synchronous avatar-mediated guidance or asynchronous reflective prompts, (c) conduct structured debriefing to bridge virtual experiences with real-world application, and (d) monitor adherence, symptom change, and risk indicators using integrated session logs and ecological momentary assessment (Ong et al., 2024).

implementation structure follows a standardized 8–12-week hybrid protocol: 30–45 min of guided immersive art per week plus 45-min telepsychology integration sessions, with progressive transfer tasks (e.g., offline creative journaling or social outreach) assigned to consolidate gains. Preliminary comparative trials suggest modest incremental benefits when immersive modules augment telepsychology, particularly in domains characterized by avoidance and motivational deficits (Hubbard et al., 2025). Mediational analyses indicate that enhanced experiential engagement, improved adherence to between-session assignments, and structured immersive debriefing may partially account for these gains (Buragohain et al., 2025). Therapist presence—synchronous via avatar-mediated participation or asynchronous through guided prompts—appears to strengthen emotional regulation and reduce attrition (Ong et al., 2024). Responsive subgroups include socially anxious individuals, patients with depressive withdrawal, and adolescents demonstrating high digital engagement (Cushnan et al., 2024). Risk management is embedded throughout. To prevent overdependence, sessions are time-limited with built-in “virtual-to-real” transfer homework; therapists track usage patterns and intervene with motivational interviewing if virtual engagement displaces offline activities. For avoidance patterns, graded real-world exposure tasks are paired with immersive rehearsal, and therapists monitor for signs of maladaptive escapism via weekly symptom and functional impairment checks (Lundin et al., 2023; Kourtesis et al., 2024).

### 8.2 Biomarkers and digital phenotyping

Physiological and behavioral markers are being explored to inform personalization and monitor engagement, although evidence remains preliminary. Electroencephalography (EEG) studies report increased frontal midline theta and modulation of posterior alpha activity during immersive engagement, patterns associated with attentional absorption and reduced self-referential processing (Castanho et al., 2025; Tong et al., 2025). However, methodological challenges—including motion artifacts, headset interference, and limited ecological validity—complicate interpretation (Kim B.-H. et al., 2025). Small sample sizes constrain external validity.

Heart rate variability (HRV) has been investigated as an index of autonomic regulation in immersive anxiety interventions (Makaroff et al., 2025). Some findings indicate transient increases in parasympathetic markers accompanying reductions in subjective anxiety, yet effects are inconsistent and typically confined to session-level changes (Kobayashi et al., 2025). Variability in recording duration, artifact correction, and analytic indices such as RMSSD versus high-frequency power limits comparability across studies (Kumpulainen et al., 2024). Evidence for sustained autonomic adaptation remains limited.

Immersive systems generate granular behavioral metrics, including gaze distribution, movement trajectories, interaction persistence, and virtual social proximity (Jung et al., 2025). Machine learning approaches—such as random forests and recurrent neural networks—have been applied to associate these digital traces with depressive and anxiety symptom trajectories (Buragohain et al., 2025). While preliminary predictive performance appears promising, small datasets increase overfitting risk and reduce generalizability (Van Mierlo et al., 2025). Importantly, exploratory correlates should not be conflated with validated clinical biomarkers. Demonstration of reliability, predictive validity, and incremental utility beyond established measures is required before integration into clinical decision-making (Torous et al., 2025). Ethical considerations include privacy intrusion, algorithmic opacity, and potential stigmatization associated with inferred risk classification (Rahangdale et al., 2025).

### 8.3 Public health implementation and regulation

Economic analyses remain limited but suggest that, once infrastructure is established, scalable immersive delivery may reduce marginal per-user costs relative to exclusively clinician-delivered therapy (Buragohain et al., 2025). However, immersive systems require greater hardware investment than telepsychology, potentially constraining implementation in low-resource settings (Wang Y. et al., 2025). Implementation structures at scale include supervised community VR stations, standardized referral pathways linked to electronic health records, and tiered clinician oversight (self-guided for low-risk cases, hybrid for moderate-risk). Barriers include equipment costs, disparities in digital literacy, clinician training requirements, and regulatory ambiguity. Public health frameworks increasingly situate immersive modules within stepped-care systems in community mental health and primary care (Cushnan et al., 2024). Integration may involve supervised VR stations, structured referral pathways, and linkage with electronic health records for longitudinal monitoring (Ong et al., 2024). Remote immersive delivery offers potential expansion into rural areas, though broadband and equipment distribution challenges persist (Buragohain et al., 2025). Large-scale monitoring typically relies on automated symptom dashboards and periodic clinician oversight to ensure safety and effectiveness (Wang Y. et al., 2025). Quality assurance standards mirror those in digital therapeutics, emphasizing usability validation, evidence generation, and post-market surveillance (Costa, 2026). Translational progression generally advances from feasibility trials to randomized efficacy studies, real-world effectiveness research, and implementation science evaluation (Raman et al., 2025).

Immersive platforms collecting behavioral and physiological data fall under regulations such as GDPR and HIPAA when deployed clinically (Halkiopoulos and Gkintoni, 2025). Safeguards include encryption, role-based access control, and explicit informed consent specifying data capture scope (Haeussl et al., 2026). Because immersive systems may record granular behavioral traces, transparency in consent is critical. Monitoring for adverse events—including cybersickness, dissociative responses, or acute distress—requires standardized protocols and clinician follow-up pathways (Hubbard et al., 2025). Structured integration of avatar-based identity exploration is recommended to prevent maladaptive detachment (Zhang and Dong, 2025). Algorithmic bias may arise from unrepresentative training datasets, necessitating transparency and equity auditing (Valente et al., 2026). In some jurisdictions, immersive therapeutic platforms qualify as regulated medical devices, requiring formal approval pathways (Dardas et al., 2025; Rajpal, 2025).

**Figure 4:**
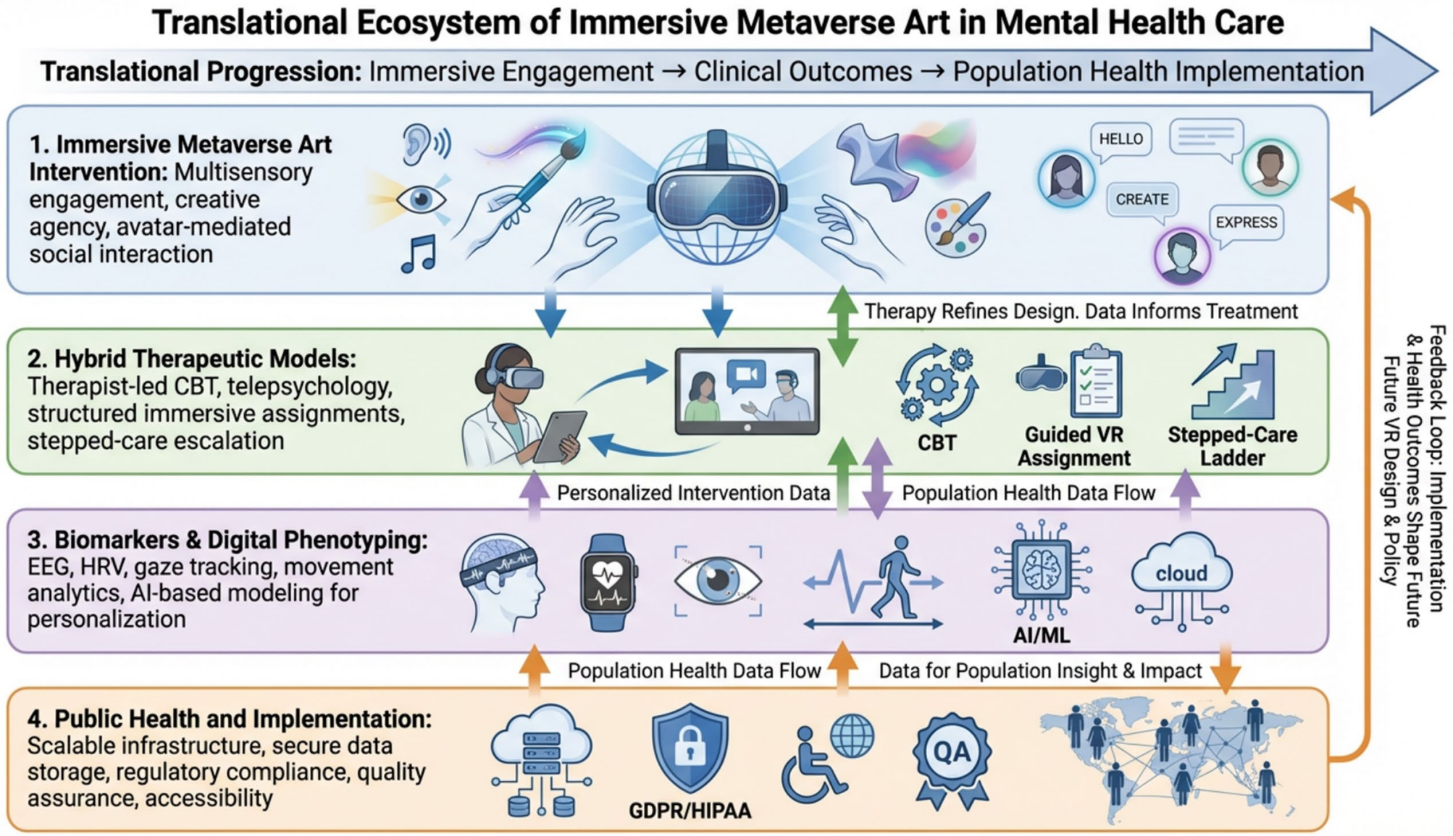
Translational ecosystem of immersive metaverse art in mental health care. The schematic illustrates the multilayered pathway from immersive artistic interventions to clinical and public health outcomes. The model integrates four interconnected layers: (1) immersive metaverse engagement, (2) hybrid clinical integration with CBT and telepsychology, (3) biomarker-informed personalization and digital phenotyping, and (4) scalable public health implementation with regulatory and infrastructure considerations. Bidirectional feedback loops highlight how clinical outcomes, biomarker insights, and implementation factors iteratively inform platform design and therapeutic deployment. This figure emphasizes mechanistic, translational, and implementation dimensions, providing a framework for integrating immersive art interventions into evidence-based mental health care.

Overall, translational advancement demands cautious integration with established therapies, rigorous validation of emerging biomarkers, scalable system design, and robust ethical governance to ensure clinically responsible implementation. Key mechanisms and translational pathways of immersive metaverse art interventions are summarized in Figure 4, highlighting their integration across clinical, biomarker, and public health domains.

## 9 Emerging technologies shaping the field

### 9.1 Artificial intelligence–personalized immersive environments

Artificial intelligence (AI) is increasingly embedded within immersive mental health platforms to enable real-time modulation of virtual environments based on inferred emotional states. Contemporary systems integrate affective computing pipelines that estimate user arousal and valence and dynamically adjust environmental parameters such as visual complexity, auditory tone, narrative pacing, and graded exposure intensity (Farsadaki and Griffy-Brown, 2026; Schlicher et al., 2025). Emotional inference is typically derived from multimodal inputs, including facial expression analysis, speech prosody, gaze tracking, movement kinematics, and physiological indices such as heart rate variability (HRV) and electrodermal activity (Zhao et al., 2026; Tabassum et al., 2025). These signals are mapped either to discrete affective categories or to dimensional valence–arousal coordinates, forming the basis for adaptive environmental calibration.

Supervised learning architectures—support vector machines, random forests, convolutional neural networks (CNNs), and long short-term memory (LSTM) networks—remain the dominant approaches for affect classification in immersive systems (Wang F. et al., 2025). More advanced implementations incorporate reinforcement learning (RL) agents that iteratively optimize adaptation policies to maximize predefined therapeutic targets, such as sustained parasympathetic activation or reduced avoidance behavior (Pathirana et al., 2024). Compared with static virtual environments, adaptive systems report higher engagement metrics and, in preliminary randomized trials, modest short-term gains in anxiety reduction and mood regulation (Dritsas and Trigka, 2026; Baber and Baker, 2025). However, small sample sizes and limited independent replication constrain confidence in these findings.

Comparative studies evaluating personalized versus non-personalized immersion commonly employ parallel-arm randomized controlled trials or micro-randomized designs isolating adaptive components (Guillen-Sanz et al., 2024). Proposed mediators include enhanced perceived autonomy, increased attentional absorption, and greater self-efficacy attributable to environmental responsiveness (Tong et al., 2025). These mechanisms align with self-determination theory, wherein personalization may strengthen intrinsic motivation by matching intervention demands to moment-to-moment affective capacity (Ernst et al., 2024). Integration with cognitive behavioral therapy (CBT) is conceptually coherent: adaptive systems can calibrate graded exposure hierarchies, detect avoidance patterns, and deliver context-sensitive cognitive prompts in response to affective markers (Wieczorek et al., 2024).

Nonetheless, algorithm-driven modulation introduces nontrivial risks. Misclassification of affective state may result in inappropriate stimulus escalation or attenuation, potentially reinforcing avoidance or dysregulation. Mitigation strategies include bounded adaptation parameters and clinician-defined safety thresholds (Khundrakpam et al., 2025). Algorithmic opacity, particularly in proprietary models lacking cross-population validation, raises concerns regarding bias and generalizability. Transparent reporting of performance metrics—accuracy, sensitivity, specificity, and calibration—along with independent replication, is essential prior to routine clinical deployment (Caragață et al., 2026).

### 9.2 Biosensor-integrated feedback systems

Biosensor integration transforms immersive environments into closed-loop biofeedback systems in which physiological signals directly influence environmental features. Interventions incorporating HRV monitors, galvanic skin response sensors, respiration belts, and electroencephalography (EEG) headsets have been evaluated primarily in stress and anxiety contexts (Lee et al., 2024; Portillo-Van Diest et al., 2024). These modalities offer objective indices of autonomic and neural regulation during immersion, although reliability varies. HRV demonstrates moderate robustness under controlled conditions but is vulnerable to motion artifacts introduced by head-mounted displays (Rafiei et al., 2025). EEG recordings face additional signal interference from movement and hardware noise, complicating extraction and interpretation (Pervez et al., 2024).

Closed-loop paradigms synchronize physiological fluctuations with adaptive environmental changes—for example, adjusting ambient lighting or auditory tempo in response to parasympathetic activation. Multimodal fusion approaches combining HRV, electrodermal activity, and behavioral indicators yield higher classification accuracy than single-modality systems (Chakrabarti, 2025). Preliminary studies suggest improved emotional regulation and engagement relative to static VR relaxation programs (Kourtesis, 2024). However, most investigations involve small samples (often n < 50), limiting statistical power and external validity.

Methodological challenges include real-time signal latency, artifact correction complexity, and heterogeneity in preprocessing pipelines. Common procedures involve bandpass filtering, independent component analysis for EEG artifact removal, and normalization relative to individual baselines (Pervez et al., 2024; Alneyadi et al., 2021). The absence of harmonized acquisition and analytic standards restricts comparability and meta-analytic synthesis. Evidence supporting long-term predictive validity of physiological metrics for sustained symptom change remains limited, underscoring the need for replication.

Continuous physiological monitoring raises ethical and governance considerations. Biological data are inherently sensitive, and ambiguity regarding ownership, secondary use, and storage duration necessitates explicit informed consent. When therapeutic claims are asserted, regulatory oversight typically aligns with digital health or medical device frameworks (Ferreira et al., 2025). Robust encryption, restricted access controls, and transparent disclosure of data handling procedures are therefore indispensable safeguards.

### 9.3 Ecological momentary assessment and longitudinal analytics

Ecological momentary assessment (EMA) is increasingly integrated into immersive systems to capture in-situ affective fluctuations and contextual responses. By collecting real-time self-reports or passive behavioral traces during and between sessions, EMA reduces retrospective recall bias inherent in conventional symptom inventories (Li Pira et al., 2023). Empirical findings indicate that momentary assessment enhances ecological validity and more accurately reflects dynamic emotional states (Catalogna et al., 2025b). Some platforms incorporate EMA-driven adaptation, modulating intervention intensity based on daily mood trajectories, with preliminary evidence of improved engagement (Buragohain et al., 2025).

Despite these advances, longitudinal studies rarely extend beyond 6 months, and follow-ups exceeding 1 year are uncommon (Lu et al., 2025b). Attrition drivers include technological fatigue, hardware constraints, and attenuation of novelty-related engagement. Behavioral immersion metrics—interaction duration, navigation variability, and exposure frequency—have been analyzed as predictors of symptom trajectories (Haeussl et al., 2026). Analytical strategies encompass multilevel modeling, autoregressive time-series analysis, and machine learning classifiers aimed at relapse detection (Halkiopoulos and Gkintoni, 2025). Although early warning models demonstrate moderate predictive performance, nontrivial false-positive rates raise concerns regarding unnecessary clinical alerts or anxiety induction (Buragohain et al., 2025).

Data governance represents a central translational challenge. Ownership of longitudinal behavioral datasets is often ambiguously defined, and predictive outputs must be communicated transparently to prevent misinterpretation. Regulatory oversight is derived from broader digital health and medical device standards (Ferreira et al., 2025), yet immersive-specific harmonization remains limited. Essential safeguards include end-to-end encryption, role-based access controls, and explicit opt-in consent for predictive analytics.

Collectively, AI personalization, biosensor-integrated feedback, and EMA-enabled longitudinal analytics reflect a convergent shift toward adaptive, data-informed immersive mental health systems. However, methodological rigor, independent replication, algorithmic transparency, and robust ethical governance remain prerequisites for responsible and sustainable clinical translation.

## 10 Future directions and research agenda

The field of immersive mental health interventions is rapidly expanding, yet substantial gaps remain in longitudinal evaluation, mechanistic understanding, cultural generalizability, and methodological standardization. Addressing these gaps is essential to establish evidence-based, scalable, and clinically relevant applications for depression, anxiety, and related affective disorders.

### 10.1 Longitudinal randomized trials

Despite promising short-term outcomes, most randomized controlled trials (RCTs) of immersive interventions focus on immediate post-session effects, with follow-ups rarely exceeding 6 months (Cheng et al., 2025; Lu et al., 2025b). Where longer-term monitoring exists, durability of symptom reduction is inconsistent, and relapse rates are seldom reported. Intervention dosage appears influential, as greater cumulative exposure correlates with sustained improvement (Lee et al., 2024), but formal dose–response studies are largely absent. Existing trials frequently involve underpowered samples, subclinical populations, or waitlist rather than active comparators, limiting mechanistic inference.

Future longitudinal RCTs should employ structurally equivalent comparators—such as non-immersive digital activities or traditional behavioral interventions—to disentangle mechanism-specific effects from general engagement or novelty-driven improvements. Retention strategies, including digital reminders, booster sessions, and hybrid therapist support, are critical to maintaining adherence. Trials must prioritize clinically diagnosed populations, account for psychiatric comorbidities through stratified or prespecified subgroup analyses, and implement extended safety monitoring for adverse events, including dissociation, acute distress, or emergent suicidality.

### 10.2 Mechanistic precision and causal testing

Mechanistic evidence for immersive interventions remains preliminary. Candidate mediators—such as reductions in rumination, enhancements in behavioral activation, attentional absorption, and social connectedness—are rarely examined longitudinally (Gardini et al., 2022). Establishing temporal precedence requires repeated measurement of mediators and outcomes across timepoints. Analytical approaches, including multilevel structural equation modeling, cross-lagged panel analyses, and dynamic mediation, are suited to uncover causal pathways (Domhardt et al., 2021). Ecological momentary assessment (EMA) offers a powerful tool to capture intra-individual fluctuations in affect, cognition, and behavior during immersive engagement. Experimental manipulation of intervention components via dismantling or factorial designs can isolate active ingredients—for example, sensory richness, narrative complexity, or social interactivity. Adaptive trial designs, in which intervention parameters adjust in response to early mediator changes, further enhance causal inference. Replication across independent samples is essential to validate mechanistic models, particularly when integrating behavioral, neural, and physiological measures.

### 10.3 Cross-cultural validation

Most immersive mental health research is conducted in Western, high-income contexts (Pedram and Piatkowski, 2025). Cultural values influence engagement with symbolic content, interpretation of narratives, and norms governing social interaction, all of which may modulate intervention efficacy. Simple linguistic translation is insufficient; interventions require culturally sensitive adaptation, ideally through participatory co-design with local stakeholders, to maximize ecological validity and acceptability. Infrastructure and digital literacy barriers in low- and middle-income countries constrain adoption and outcomes. Outcome measures must demonstrate cross-cultural measurement invariance, ensuring that symptom and process-based instruments (e.g., behavioral activation, social connectedness) function equivalently across populations. Multisite cross-cultural trials incorporating participatory design principles are necessary to establish generalizable efficacy and identify culturally specific moderators.

### 10.4 Standardized outcome metrics

Heterogeneity in outcome reporting limits synthesis and cumulative evidence building. Common symptom measures include PHQ-9, BDI-II, GAD-7, and STAI (Zeng et al., 2025), yet process-based constructs central to immersive intervention theory—rumination, attentional absorption, and sense of belonging—are underreported. Minimum reporting standards should encompass validated symptom and process measures, adverse events, and session-level engagement metrics.

Physiological markers (e.g., HRV) and digital behavioral metrics require standardized acquisition, preprocessing, and reporting to ensure interpretability. Clinically meaningful change should be defined through reliable change indices or established minimal clinically important differences. Preregistration, open datasets, and harmonized data formats will facilitate replication, meta-analysis, and cumulative knowledge development.

### 10.5 Interdisciplinary framework development

Advancing immersive mental health interventions necessitates integration across clinical psychology, neuroscience, consumer experience research, and digital design. Process-based therapeutic frameworks provide a conceptual scaffold situating immersive interventions within transdiagnostic mechanisms (Li Pira et al., 2023). Collaborative partnerships between clinicians and technologists enhance both therapeutic validity and technological sophistication.

Hybrid effectiveness–implementation study designs facilitate simultaneous evaluation of efficacy, feasibility, and scalability. Early incorporation of health economic analyses informs cost-effectiveness and potential reimbursement strategies. Professional training standards for clinicians implementing immersive platforms are critical to ensure competence in safety monitoring, ethical practice, and technical operation. Coordinated multicenter networks reduce research fragmentation, support replication, and enable cross-cultural generalizability. Governance structures must balance ethical oversight with innovation, promoting transparent reporting, participant safety, and responsible data management.

In summary, the field requires rigorous longitudinal RCTs, precise mechanistic investigations, cross-cultural validation, standardized outcome metrics, and structured interdisciplinary collaboration. Addressing these priorities will consolidate immersive mental health interventions as scientifically credible, scalable, and clinically relevant tools for the treatment of depression, anxiety, and related conditions.

## 11 Conclusion

The evidence to date suggests that immersive metaverse art holds considerable potential as a novel mental health intervention. Studies of VR art experiences consistently report acute emotional benefits: for example, participants in immersive art installations experience significant reductions in negative affect and increases in positive mood. Similarly, active creation in VR (e.g., virtual sculpting) has been shown to lower anxiety levels and enhance well-being in young adults. These findings align with broader literature showing that art engagement activates reward and motivation circuits and can foster self-reflection and resilience. Immersive art, by amplifying engagement through multisensory stimuli, seems to multiply these effects: users often report profound states of flow, awe, or relaxation that exceed those of traditional media. Our multilevel model ties together these effects. Core elements like presence, creative agency, avatar embodiment, and cultural-symbolic content work together to drive outcomes. For instance, a user who designs an avatar that reflects their cultural heritage may feel more connected and invested in the experience, enhancing a sense of belonging and meaning. According to the Proteus effect and related findings, such avatar customization can improve user engagement and even therapeutic benefit. These psychological mediators – flow, identity experimentation, social connection – then translate into downstream changes: reductions in rumination, increased behavioral activation (through creative tasks), and improved emotional regulation. In clinical translation, these pathways receive concrete operationalization. Engagement-focused protocols target rumination via flow-inducing artistic tasks with therapist-guided debriefing; identity-focused protocols address social avoidance through avatar-mediated collaborative creation, followed by structured transfer to offline interactions. Therapists select patients using explicit criteria (moderate symptom severity, digital literacy, low dissociation risk), deliver hybrid 8–12-week programs, and implement risk-management protocols (session limits, offline homework, ongoing monitoring of overdependence or avoidance) to ensure safety and generalization. In practice, this could look like a depressed person discovering new self-efficacy by successfully completing a VR art puzzle, or an anxious individual finding comfort in a virtual community space with others who share their cultural symbols.

Comparatively, immersive art therapy shares some common ground with traditional art therapy (e.g., both use creativity to express emotion), but it offers unique advantages. It is inherently scalable (software can reach many users) and highly personalized (users can tailor environments and avatars to their preferences). Importantly, it can appeal to those who shy away from conventional therapy: the gaming-like nature and sense of anonymity can lower stigma and increase motivation. On the other hand, the novelty of the medium raises issues. Placebo effects and the “wow factor” of VR may inflate early results; disentangling true therapeutic action from novelty will require careful controls. There are also risks: unlimited escapism or dependence on virtual experiences could potentially worsen real-world withdrawal for vulnerable users. Some studies caution that heavy metaverse use can be associated with anxiety, depressive symptoms, or addictive patterns. These concerns underscore the need for ethical guidelines and balanced use.

Looking ahead, the trajectory of this field is both exciting and uncertain. Technological trends – AI-driven personalization, biosensor feedback, and continuous tracking – promise even richer interventions. For example, adaptive VR environments might tailor their difficulty or emotional tone in real-time to a user’s stress level. Integrating VR art programs with established therapies (e.g., as an adjunct to CBT homework or telepsychology) could create powerful hybrid models. Crucially, the literature repeatedly calls for rigorous research designs. Most existing studies are small or exploratory; well-powered longitudinal trials are needed to confirm efficacy and durability. We must also develop standardized outcome measures (beyond self-report) and identify relevant biomarkers or digital phenotypes. Finally, equity and cultural access are paramount: efforts should ensure that immersive art interventions are inclusive, culturally sensitive, and distributed without exacerbating the digital divide.

In conclusion, immersive metaverse art represents a multidisciplinary frontier at the intersection of consumer technology, cultural psychology, and mental health. It offers a novel mode of engagement that can modulate mood, identity, and cognition in positive ways. While still early in development, the accumulating evidence – from mood improvements in pilots to converging theoretical insights – suggests this approach is more than a passing fad. If thoughtfully designed and rigorously tested, VR art interventions could become a valuable complement to traditional therapies, expanding the toolbox for addressing depression and anxiety in our digital age. With the concrete clinical pathways, therapist protocols, patient-selection criteria, implementation structures, and risk-management safeguards now delineated, VR art interventions—if thoughtfully designed and rigorously tested—could become a valuable complement to traditional therapies, expanding the toolbox for addressing depression and anxiety in our digital age.
